# The interplay of early-life stress, nutrition, and immune activation programs adult hippocampal structure and function

**DOI:** 10.3389/fnmol.2014.00103

**Published:** 2015-01-09

**Authors:** Lianne Hoeijmakers, Paul J. Lucassen, Aniko Korosi

**Affiliations:** Center for Neuroscience, Swammerdam Institute for Life Sciences, University of AmsterdamAmsterdam, Netherlands

**Keywords:** hippocampus, neurogenesis, cognition, early-life stress, early-life nutrition, early-life neuroimmune activation, early-life infection

## Abstract

Early-life adversity increases the vulnerability to develop psychopathologies and cognitive decline later in life. This association is supported by clinical and preclinical studies. Remarkably, experiences of stress during this sensitive period, in the form of abuse or neglect but also early malnutrition or an early immune challenge elicit very similar long-term effects on brain structure and function. During early-life, both exogenous factors like nutrition and maternal care, as well as endogenous modulators, including stress hormones and mediator of immunological activity affect brain development. The interplay of these key elements and their underlying molecular mechanisms are not fully understood. We discuss here the hypothesis that exposure to early-life adversity (specifically stress, under/malnutrition and infection) leads to life-long alterations in hippocampal-related cognitive functions, at least partly via changes in hippocampal neurogenesis. We further discuss how these different key elements of the early-life environment interact and affect one another and suggest that it is a synergistic action of these elements that shapes cognition throughout life. Finally, we consider different intervention studies aiming to prevent these early-life adversity induced consequences. The emerging evidence for the intriguing interplay of stress, nutrition, and immune activity in the early-life programming calls for a more in depth understanding of the interaction of these elements and the underlying mechanisms. This knowledge will help to develop intervention strategies that will converge on a more complete set of changes induced by early-life adversity.

## INTRODUCTION

Clinical studies have provided evidence that cognition in later life is strongly influenced by experiences occurring during the sensitive period of early development and adolescence. Indeed, adverse early-life events, e.g., social deprivation or abuse, are associated with an increased vulnerability to develop psychiatric disorders (Stevens et al., 2008; Maselko et al., 2011) and impaired cognitive functioning in adulthood (Chugani et al., 2001; Kaplan et al., 2001; Nelson et al., 2007; Mueller et al., 2010). Interestingly, exposure to prenatal or postnatal malnutrition, e.g., the lack of one or multiple essential nutrients, can lead to a similarly increased incidence of psychopathologies (Brown et al., 2000; Costello et al., 2007; Mueller et al., 2010) and cognitive deficits in adolescence and adulthood (Walker et al., 2000; Benton, 2010; de Rooij et al., 2010; de Groot et al., 2011; de Souza et al., 2011; Laus et al., 2011).

Different lines of work further illustrate the relation between cognitive functions and postnatal immune system activity. For example, maternal inflammatory responses during pregnancy (Eriksen et al., 2009), antenatal infection in preterm babies (Dammann et al., 2002; Eriksen et al., 2009; van der Ree et al., 2011) and neonatal infection (Rantakallio et al., 1997; Libbey et al., 2005) are suggested as risk factors for a lower IQ later in life and for an increased vulnerability to develop later neuropsychiatric disorders like schizophrenia and depression (Brown, 2006; Cope and Gould, 2013; Miller et al., 2013; O’Connor et al., 2014). These lines of research support the hypothesis postulated in the developmental origins theory of Barker that the early-life environment determines the framework for later adult functioning and the development of pathologies through an early programming of adult brain structure and function (Barker, 2004; Barker et al., 2013). Although the consequences of an adverse early-life history for adult cognitive function are confirmed by evidence from preclinical studies in rodents (Lucassen et al., 2013; Naninck et al., 2013), the exact mechanisms underlying these programming effects and the determining elements in the environment that modulate these processes remain obscure. As is clear from the above examples, early-life environmental experiences cannot be avoided (Danese et al., 2009; Bale et al., 2010) and it is thus crucial to understand which elements play a role, and how they interact in order to develop future interventions.

The early postnatal environment encompasses many essential elements, which are key and determinant for proper brain development, many of which are largely transmitted via the mother–child interaction. A child is generally dependent on maternal care during the first weeks of life, encompassing tactile stimulation, nutritional provision, as well as transfer of antibodies and maternal warmth. As is evident from the above examples, an adverse early-life environment may affect the stress hormones, nutrition, or inflammatory modulators, all elements that can strongly interact and affect one another (Kelly and Coutts, 2000; Shanks and Lightman, 2001; Miller et al., 2009; Palmer, 2011). Hence, an individual is actually exposed to a combination of these factors rather than to one of these elements independently.

There is, e.g., increasing evidence that early-life maltreatment is associated with an increase in the pro-inflammatory markers of the immune system in adulthood (Coelho et al., 2014). Also, associations have been found between maternal supplementation of specific nutrients (e.g., folate, iodine, and vitamin D) and an enhanced fetal immune system development that was paralleled by a reduced incidence of psychopathologies in adulthood (Marques et al., 2013). Also, Monk et al. (2013) reviewed the interrelation of nutrition and prenatal stress in stress-induced maternal malnutrition. An interrelated profile of different early-life elements brings forward the possibility of confounding factors in the currently reported findings on the possible mechanisms that underlie such programming. Interestingly, most studies addressing the mechanisms underlying early-life programming consider these elements individually, but, considering that each one of these elements has a very strong interaction with one another, and influence each other greatly, it is likely that the final effect will be determined by the synergistic action of the different early-life elements at play. Hence, it will be necessary to re-discuss and re-analyze the so far obtained results in light of such interactions.

The focus of this review will be on the essential elements present in the early postnatal environment and their involvement in the lasting effects on cognitive functioning. In particular we will discuss how the various elements during the early postnatal period (i.e., sensory stimuli, nutrition, stress hormones, and inflammatory molecules) interact, affect each other and ultimately how they may synergistically affect brain structure, and function on the long term. We focus on the consequences for hippocampal structure and related cognitive functioning and on a unique form of hippocampal plasticity, adult neurogenesis. The hippocampus is one of the key brain regions important for cognitive functions and this form of plasticity is very important for learning and memory processes and highly modulated by (early) environmental factors, i.e., stress (Gould et al., 2000; Mirescu et al., 2004; Dranovsky and Hen, 2006; Lucassen et al., 2013), nutrition (Lindqvist et al., 2006; Beltz et al., 2007; Coupé et al., 2009), and immune activation (Das and Basu, 2008; Green and Nolan, 2014; Musaelyan et al., 2014).

The fact that the hippocampus is particularly susceptible to influences of the early-life environment and in particular stressful stimuli is easily understood when considering the important developmental processes that take place in this brain region during this sensitive developmental period. Indeed, hippocampal and dentate gyrus (DG) development in particular starts during late gestation and continues during the first 2 weeks after birth (Altman and Bayer, 1990), while in human the development of the DG starts during the last trimester of pregnancy and continues to about 16 years of age (Arnold and Trojanowski, 1996). During this time, granular cells are generated in the subventricular zone or in the hippocampus itself, that migrate to the different layers of the DG (Pleasure et al., 2000; Fukuda et al., 2005; Navarro-Quiroga et al., 2006), while in adulthood quiescent neuronal progenitors cells develop to become functional granular cells (Kempermann et al., 2004). Interestingly, adult neurogenesis is lastingly affected by perturbation of the early-life environment as well (Coupé et al., 2009; Oomen et al., 2011; Korosi et al., 2012; Lucassen et al., 2013; Loi et al., 2014; Musaelyan et al., 2014). Next to the generation and migration of granular cells, the migration and colonization by microglia takes place as well during this sensitive period and is also peeking in the first few postnatal days (Schwarz and Bilbo, 2012; Schwarz et al., 2012; Cope and Gould, 2013). These migratory processes are supported by a scaffold formed by immature astrocytes. In addition, glia cells are increasingly acknowledged for there role in the plasticity and circuit functioning of the adult hippocampus (Allen and Barres, 2009; Ekdahl, 2012). Finally, the hippocampus is highly sensitive to stress both early as well as during adult life due to its remarkably high expression levels of the glucocorticoid receptor (GR). Interestingly, expression levels of these receptors have been shown to be affected by early-life stress as well (Liu et al., 1997; Lucassen et al., 2013; de Kloet et al., 2014).

In the following sections we discuss the effects of early-life stress, nutrition, and central immune activity on hippocampal function and adult neurogenesis and thereafter discuss how to implement in these findings that (1) these elements affect one another and (2) they act synergistically to exert their function.

## MODULATION OF HIPPOCAMPAL FUNCTION AND NEUROGENESIS BY EARLY-LIFE STRESS

Early-life stress exposure is strongly associated with cognitive impairments later in life (Kaplan et al., 2001; Stevens et al., 2008; Mueller et al., 2010; Maselko et al., 2011), but what is the preclinical evidence for this association? Which are the most common animal models to study this question?

Stress in the postnatal period can be induced using several rodent paradigms. The most widely studied models to induce early-life stress involve either naturally occurring variation or artificial modulation of maternal care (Francis and Meaney, 1999; Francis et al., 2000), repeated dam-litter separation or a single prolonged deprivation of the dam and her pups (Schmidt et al., 2010; Hedges and Woon, 2011) and chronic early-life stress (Ivy et al., 2008; Rice et al., 2008; Wang et al., 2011) during the first few postnatal weeks. Adult offspring from all of these early-life stress models exhibit cognitive impairments, indicating a strong translational value of these models in addressing the underlying mechanism of such programming. For example, adult rats that had been either maternally separated during the first 2 weeks of life or deprived on postnatal day (P)3 for 24 h, exhibited impairments in their acquisition of spatial information in the Morris water maze (MWM; Oitzl et al., 2000; Huot et al., 2002; Aisa et al., 2007; Fabricius et al., 2008; Oomen et al., 2010) and mice exposed to chronic early-life stress show impairment of spatial memory (tested by MWM and object location) and declarative memory tested by novel object recognition task and Y-maze (Rice et al., 2008; Wang et al., 2011). These early-life stress induced cognitive impairments are associated with a number of alterations in hippocampal structure and neuronal plasticity, including decrease in dendritic complexity and spine density in the cornu ammonis (CA)1 and CA3 (Huot et al., 2002; Ivy et al., 2008; Wang et al., 2011), reduced DG dendritic complexity, granular cell number and granular cell density (Oomen et al., 2010, 2011), reduced astrocyte density in the DG and CA regions (Leventopoulos et al., 2007), and age-dependent alterations in adult hippocampal neurogenesis levels (Korosi et al., 2012). Offspring in both rats and mice exposed to a form of early-life stress exhibit a short-term increase followed by a permanent reduction of proliferating and immature cells (Mirescu et al., 2004; Nair et al., 2007; Oomen et al., 2009, 2010; Hulshof et al., 2011; Suri et al., 2013; Naninck et al., 2014). Levels of cell survival and astrogenesis in young adults are generally not affected after maternal separation (Oomen et al., 2010; Hulshof et al., 2011; Kumar et al., 2011; Suri et al., 2013), but this is strongly reduced at a more advanced age both in rats exposed to low levels of maternal care (Bredy et al., 2003), as well as in mice exposed to chronic early-life stress (Naninck et al., 2014).

How exactly does early-life stress exposure lead to the above described lasting alterations in cognitive functions as well as hippocampal structure and function remains relatively uncertain, but several possible mechanisms of action have been identified over the last years. First, available evidence that early-life stress manipulations, including maternal separation, maternal deprivation or chronic stress via environmental manipulation, alter the quality and/or quantity of maternal care (Brown et al., 1977; Pryce et al., 2001; Macrì et al., 2004; Brunson, 2005; Fenoglio et al., 2006; Korosi and Baram, 2010) strongly suggests that mother–infant interaction is crucial in programming brain and behavior. However, these studies do not directly address whether maternal care, under normal conditions, is actively involved in these regulations.

In support of this notion, it has been demonstrated that a natural variation in maternal care (Liu et al., 1997; Champagne et al., 2008; Bagot et al., 2009) and individual within-litter variation in the amount of active care received (van Hasselt et al., 2012a,b) leads to differences in stress response and cognitive functions associated with altered hippocampal structure, plasticity and changes in the neuroendocrine system in later life. In line with this evidence from animal studies, in pre-term and term neonates, different forms of sensory stimulation, such as moderate touch or skin-to-skin care, have been shown to have beneficial consequences, including reduction of pain responsiveness in neonates (Cignacco et al., 2007) and a reduced reactivity to stress (Feldman et al., 2010). While it is clear that sensory stimuli that the mother gives to her offspring is highly dependent on the well-being of the mother and offspring and thus strongly affected by stressful environment, how exactly this element interacts with the other key elements in the early environment to lead to the programming of the brain structure and function is yet unclear. The few studies tackling the interaction of sensory stimuli with either nutritional or immune challenges are discussed in Sections “The Interplay of the Different Elements in the Early-Life Environment” and “Early-Life Adversity; Opportunities for Intervention Later in Life.”

The role of stress-related hormones (corticosterone; CORT) and neuropeptides (e.g., corticotropin releasing hormone, CRH) in the modulation of early-life stress effects on the hippocampus has been studied extensively. When the HPA axis is activated by a stressor this leads to HPA axis activation, which in turn leads to the initial release of CRH from the hypothalamic paraventricular nucleus (PVN), stimulation of pituitary adrenocorticotropic hormone (ACTH) secretion into the blood, and the subsequent release of glucocorticoids from the adrenal glands: CORT in rodents and cortisol in humans. Negative feedback takes place when glucocorticoids bind to GRs in the hippocampus, PVN, prefrontal cortex and pituitary and thereby inhibit release of CRH and ACTH (Tsigos and Chrousos, 2002).

In fact, exposure to stress during the postnatal early-life period programs the basal and stress-induced activation of the HPA axis and the behavioral responses to stress throughout life (Seckl and Meaney, 2006; Heim and Binder, 2012). Importantly during the first 2 weeks of life, the stress response is believed to be hypo-responsive to some, but not all stressors. This consists of a smaller, or absence of HPA axis responsiveness in pups when compared to the adult organism (Stanton et al., 1988; Levine et al., 1991). Age appropriate stressors, like maternal deprivation or fragmented maternal care elicit secretion of CORT (Yi and Baram, 1994; Schmidt et al., 2004; Rice et al., 2008). There is increasing evidence that next to an increased release of CORT (basally and upon stress exposure), the expression levels of several of the genes involved in the modulation of the stress response (e.g., CRH and GR; Rice et al., 2008; Ivy et al., 2010; Wang et al., 2011; Chen et al., 2012) are lastingly altered in offspring experiencing early-life stress. Thus it is reasonable to assume that these changes might be (at least partly) mediating the altered hippocampal plasticity and thereby the associated cognitive impairments. However, the lasting alterations in the levels of circulating CORT are not consistently permanent in different models of early-life stress (Mirescu et al., 2004; Brunson, 2005; Rice et al., 2008).

While glucocorticoid exposure during early-life evoked cognitive impairments in adulthood (Kamphuis et al., 2003) and there is abundant literature about the regulating (mostly inhibiting) role of CORT (during adulthood) on neurogenesis (Cameron and Gould, 1994; Lucassen et al., 2010), chronic depletion of CORT through adrenalectomy of rats at 10 days of age did, however, not induce alterations in neurogenesis in adulthood (Brunson et al., 2005). Whether the raise in CORT early in life (and no longer in adulthood) modulates the process of neurogenesis and cognitive impairments on the long-term thus remains to be determined. The paucity of data points to the need for further research in this area; however, the contradictory data from the existing studies suggest that other factors may also contribute to the mechanisms by which early-life experience programs brain structure and function.

Because CRH expression is permanently altered after early stress in various models in the hypothalamus (Plotsky and Meaney, 1993; Liu et al., 1997) and hippocampus (Ivy et al., 2010), CRH has been explored as a modulator for the consequence of early-life stress in the hippocampus (Brunson, 2005; Ivy et al., 2010; Korosi et al., 2010; Wang et al., 2011; Loi et al., 2014). Indeed, exposure to CRH can mimic the changes in hippocampal structure induced after chronic early-life stress (Brunson, 2005) and a selective blockage of the CRF receptor type 1 during the first week after chronic early-life stress indeed prevented the apparent cognitive impairments in the early-life stressed animals (Ivy et al., 2010). Intriguingly, conditional CRF1 knock-out mice were ‘protected’ against the hippocampus dependent cognitive impairments induced by chronic early-life stress (Wang et al., 2011). Finally early-life stress has been shown to age-dependently affect the expression of the gene and protein of the neurogenic factor brain derived neurotrophic factor (BDNF; Nair et al., 2007; Zimmerberg et al., 2009; Suri et al., 2013). Indeed BDNF expression and concomitant levels of hippocampal neurogenesis are upregulated by early-life stress during development and young adulthood, but reduced to decreased levels with aging (Nair et al., 2007; Suri et al., 2013; Naninck et al., 2014). These data suggests a possible role of BDNF in the modulation of hippocampal plasticity after early-life adversities.

Summarizing, stress related hormones and neuropeptides are involved in mediating lasting effects of early-life stress, however they are not sufficient to explain all the observed effects, indicating that other factors, possibly acting synergistically with these stress molecules, are also involved in the programming. We will next explore the role of nutrition early in life in these processes.

## EARLY NUTRITIONAL FACTORS DETERMINE ADULT HIPPOCAMPAL STRUCTURE AND FUNCTION

As mentioned in the introduction, early nutritional insults have lasting consequences for brain development and function later in life (Lucas, 1998; Brown et al., 2000; McMillen et al., 2008; Prado and Dewey, 2014) with later cognitive functions being particularly affected (de Groot et al., 2011). This is not surprising when considering that during the first postnatal period, the brain is under heavy development and an incredible nutritional demand. In fact, for proper brain development to occur, specific dietary macro-, and micronutrients are essential during gestation and lactation (Scholl et al., 1996; Benton, 2010; Dangat et al., 2010; Veena et al., 2010). Thus, disruption of the nutritional supply (quality and quantity) to the offspring will have major effects on the development of the brain, and more specifically on the hippocampus. An inadequate supply of them during critical developmental periods leads to brain dysfunction and cognitive impairments later in life (McNamara and Carlson, 2006; Innis, 2008; de Groot et al., 2011; Laus et al., 2011).

The offspring is during this critical developmental period fully dependent on the nutrition provided by the mother. Most preclinical models are based upon altering maternal nutrition during gestation and/or lactation. Indeed, micronutrient composition of the maternal diet during gestation and lactation determine the balancing of fatty acid (FA) levels in the brain of the offspring, as maternal micronutrients (Roy et al., 2012) affect the breast milk composition (Innis, 2008; Allen, 2012). The association of early-life nutrition and cognitive functions is further supported by preclinical evidence (Campbell and Bedi, 1989; Castro et al., 1989; de Souza et al., 2011; Valladolid-Acebes et al., 2011; André et al., 2014). Here, we will discuss how early postnatal nutritional stress and specific nutritional components present in the postnatal period modulate cognition and neurogenesis in adulthood.

Various models are used to study how early malnourishment affects brain development and cognitive functions, e.g., through dietary restriction, overnutrition, or malnutrition by limitation of different key elements during gestation and/or lactation. For instance, protein restriction, global dietary restriction to 50% or high-fat, and modulation of essential macro- and micronutrients that need to be obtained from the diet are commonly used approaches (Campbell and Bedi, 1989; Bedi, 1992; Martinez et al., 2009; Valadares et al., 2010; de Souza et al., 2011; Roy et al., 2012). For example, protein restriction during lactation in rats (Valadares et al., 2010) and 12 h restriction of maternal milk (Castro et al., 1989) evokes deficits in hippocampus dependent spatial memory tested by MWM and object recognition in adult offspring, but see (Wolf et al., 1986; Campbell and Bedi, 1989). These deficits are accompanied by alterations in hippocampal structure and plasticity as well. Food restriction to 50% of the normal intake during lactation changed the time course of BDNF production and proliferation in the hippocampus (Coupé et al., 2009) and reduced the number of proliferating cells in the adult offspring, without affecting cell survival or cell fate (Matos et al., 2011). Furthermore, protein restriction during the same period leads to reduced total granular cell numbers (Bedi, 1991) and food restriction to 50% during gestation and lactation reduced hippocampal volume (Katz et al., 1982). In addition to these nutritional restriction studies, the offspring of high-fat diet exposed dams exhibit a reduction in postnatal neurogenesis during development (Tozuka et al., 2009) and impaired dendritic differentiation of newborn neurons in the adult hippocampus (Tozuka et al., 2010). However, to date no further studies of adult hippocampal neurogenesis in early-life food-restricted or high-fat diet exposed animals have been performed.

The lipid content during early-life is essential for the composition of maternal milk during lactation and development of the pup brain. For instance, polyunsaturated fatty acids (PUFAs), including the omega-3 FA docosahexaenoic acid (DHA) and omega-6 FA arachidonic acid (AA), are structural components of the brain that promote healthy neuronal growth, repair, and myelination (McNamara and Carlson, 2006). Deficiency of these FAs in the maternal diet first revealed the association of low FA composition and impaired learning and memory functions (Lamptey and Walker, 1978). Moreover, deficiency of omega-3 FA during gestation and lactation impairs the spatial memory (tested by Barnes maze; Fedorova et al., 2009), whereas artificial feeding of rats with omega-3 FA deficient milk during lactation prolonged escape latency in the MWM (Lim et al., 2005) and omega-3 FA enrichment improved performance of the animals (Carrié et al., 2000).

These functional changes following FA deficiency are furthermore associated with structural changes in the brain. Maternal omega-3 FA deficiency during gestation leads to underdevelopment of the primordial hippocampus in fetal rats at the last days of gestation (Bertrand et al., 2006). Nutritional omega-3 FA deficiency during gestation and lactation reduces pyramidal cell size in the hippocampus (Ahmad et al., 2002) and the levels of markers for neuronal plasticity such as BDNF (Madore et al., 2014) at weaning. In addition, dietary enrichment with omega-3 prevents the adverse consequences of early-life sevoflurane (anesthesia) exposure on cell proliferation in the hippocampus and the induced memory impairments (Lei et al., 2013). Maternal supplementation of α-linolenic acid (ALA), a precursor of omega-3 FA, during gestation and lactation enhanced hippocampal neurogenesis at P19 (Niculescu et al., 2011). However, to date it has not been studied whether an imbalance of FAs in early-life lastingly affects adult hippocampal neurogenesis.

Next to essential FAs, essential amino acids, choline, and methionine, and micronutrients such as folic acid (B9), vitamin B6 and B12, are also essential for brain development (Roy et al., 2012). Deficiency of choline during gestation and lactation impairs working memory in the 12-arm spatial memory maze, while supplementation enhances performance (Meck and Williams, 1999). In addition, a deficiency of nutritional folate, choline, B6, and B12 during gestation and lactation leads to learning and memory impairments in the radial arm mazes and enhanced the number of apoptotic cells in the hippocampus (Blaise et al., 2007). Deficiency of these methyl donors furthermore affects hippocampal neurogenesis by altering the apoptotic rate (Craciunescu et al., 2010).

Summarizing, evidently the nutritional composition during critical developmental periods (pre and postnatal) of life is essential for the proper development, structure and function of the hippocampus. Similar to the cognitive impairments induced by early-life stress, early-life malnutrition evokes such deficits as well. Another important element known to play a key role in modulating brain development and function is the neuroimmune system, which will be discussed in the next section.

## EARLY-IMMUNE RESPONSE ACTIVATION PROGRAMMING THE LATER-LIFE HIPPOCAMPUS

Activation of the peripheral and/or central immune system in early-life is associated with psychopathologies in adulthood, including cognitive dysfunction (Rantakallio et al., 1997; O’Connor et al., 2014). For instance, maternal infection during pregnancy is associated with lower IQ in adult men (Eriksen et al., 2009). In addition, pre- and postnatal infection have been associated with anxiety-like and depressive-like behavioral responses and cognitive impairments in adolescence and adulthood (Das and Basu, 2011; Williamson et al., 2011; Doosti et al., 2013; Dinel et al., 2014). The modulating effects of early-life immune challenges on brain function are not unexpected considering the essential role of neuroimmune cells in (early-)life. Microglia and astrocytes mediate many processes in the brain, including neuroinflammatory responses (Capuron and Miller, 2011; Green and Nolan, 2014; O’Connor et al., 2014), neuronal activation and plasticity (Slezak et al., 2006; Halassa et al., 2009; Parpura et al., 2012; Greter and Merad, 2013), maintenance and development of the blood–brain barrier (BBB; Banks et al., 1995; Chaboub and Deneen, 2013) and importantly, neurogenic processes during development (Das and Basu, 2011; Schwarz and Bilbo, 2012; Cope and Gould, 2013) and adulthood (Das and Basu, 2008; Ekdahl, 2012; Cope and Gould, 2013; Cunningham et al., 2013; Kohman and Rhodes, 2013; Sierra et al., 2013, 2014). Thus, imbalanced activation of the microglia, in particular during early-life, has the potential to lastingly disturb internal homeostasis and proper brain development (Allen and Barres, 2009).

Activity of the microglia is controlled by immune response regulating effector molecules, like pro-inflammatory (e.g., IL-1β, IL-6, and TNFα) and anti-inflammatory (e.g., IL-4 and IL-10) cytokines or chemokines (Chaplin, 2003; Cartier et al., 2005; Ekdahl et al., 2009) that regulate the communication between immune cells in the peripheral and central immune system. Although the brain is a relatively concealed and immunosuppressed environment in adulthood, that is separated from the periphery by the BBB, cytokines, and chemokines in the periphery have the potential to cross the BBB and can affect the innate cells of the brain (Banks et al., 1995; Pan and Kastin, 1999). Interestingly, during early-life, peripheral immune challenges might have a greater potential to adversely affect the brain (Schoderboeck et al., 2009). During this time, the BBB still pre-exists in a leaky stage till a few days after birth (Engelhardt, 2003), providing the possibility of a greater immunoreactive responses in the brain when a peripheral infection occurs. In addition, microglia develop in close parallel to developmental neurogenesis and appear in an activate and amoeboid state during development, whereas they are present as resting, ramified cells in adulthood (Bilbo and Schwarz, 2009).

In the following part, we will discuss the pre-clinical evidence in support of a direct role of postnatal immune challenges in the persistent modulation of hippocampal structure and function. Most studies of postnatal infection have focused on stimulation by bacteria like *Escherichia coli* or the Gram-negative bacteria component lipopolysaccharide (LPS). We will here address the hippocampus dependent cognitive functions following early-life neuroimmune stress from two different angles. Firstly, activation of the peripheral neuroimmune system and its immediate and lasting effects on central neuroimmune system function and brain function. Secondly, the consequences of central neuroimmune system activity without a prior peripheral immune challenge, for instance via activation of central viral infection or pro-inflammatory factors, on hippocampal function and neurogenesis in adulthood.

A peripheral immune challenge with LPS (P1) or *E. coli* (P4) in the rat pup elicits an elevation of pro-inflammatory cytokines and CORT in the first few hours after the challenge in blood serum (Bilbo et al., 2005b), whole brain (Ortega et al., 2011) or hippocampus (Bilbo et al., 2005b; Dinel et al., 2014). In adulthood, cytokine mRNA expression levels in the brain of the early-infected animals remain normal under basal conditions, with the exception of elevated hippocampal levels of TNFα (P4 infected; Bland et al., 2010a) and IL-1β (P5 LPS infected; Wang et al., 2013). Although overall no strong changes in the cytokine expression profiles are present in the adult, in the early-life infected animals, microglial activation markers indicate enhanced reactive microglia (CD11b+) in the hippocampus (Bilbo et al., 2005a) and CA1 region (Iba1+; Bland et al., 2010b). In addition, adult rats exposed to LPS at P3 and P5 exhibited a hippocampus-specific increase in Iba1+ immunoreactive microglia in the CA1 and DG (Sominsky et al., 2012), indicative of a priming effect on hippocampal microglia. Indeed, a peripheral LPS injection in adulthood exerts an exaggerated pro-inflammatory cytokine response of mainly IL-1β in the hippocampal CA1 of rats with a history of early-life infection (Bilbo et al., 2005a, 2008), probably evoked by a programmed pro-inflammatory response of hippocampal microglia.

Interestingly, the pro-inflammatory response in the hippocampus following peripheral infection is accompanied by a direct effect on developmental hippocampal neurogenesis and structural changes in adulthood. *E. coli* infection at P4 immediately suppresses gene expression of neurotropic factor BDNF in the CA1 and CA3 (Bilbo et al., 2008) and neuronal and astrocytic cell proliferation is reduced in the hippocampus following LPS exposure at P9 (Järlestedt et al., 2013). Hippocampal cell proliferation then restores to normal conditions in the 48 h after the challenge and does not affect the survival of immature neurons during the time of infection (Bland et al., 2010a; Järlestedt et al., 2013). This developmental change probably underlies the reduction in later hippocampal volume observed in early-life infected adult rodents (Wang et al., 2013). Moreover, in adulthood, early-life *E. coli* infected rats and LPS infected mice have comparable numbers of dividing, differentiating, and surviving neurogenic cells in the subgranular zone as control animals (Bland et al., 2010b; Järlestedt et al., 2013; Dinel et al., 2014). In conclusion, neurogenesis does not seem to be heavily affected by peripheral immune challenges. These subtle changes in neurogenesis are in line with the findings of limited effects on hippocampus-related cognitive functioning as well. Most studies of early-life infection do not find changes in different learning and memory paradigms, such as fear conditioning, MWM or Y-maze (Bilbo et al., 2005a, 2007, 2008; Dinel et al., 2014), but see (Harré et al., 2008; Wang et al., 2013). Interestingly, however, a stronger modulation of hippocampus-related cognitive functions is manifested after a second immunological challenge in early-life infected rodents. Thus, a combination of early-life infection history and adolescent or adult LPS (re-)exposure evokes impairments in a contextual fear conditioning paradigm (Bilbo et al., 2005a, 2006) and spatial memory performance in the Y-maze (Dinel et al., 2014), but not in the MWM (Bilbo et al., 2007).

Interestingly, these cognitive impairments in response to a second immune challenge appear in accordance with reduced newborn cell survival in the early-infected rats (Bland et al., 2010a), but not with BDNF gene expression levels (Bilbo et al., 2008). In contrast, neurogenesis is upregulated after LPS during adulthood in animals that never had an immune challenge before (Bland et al., 2010a). The consequences of a second immunological challenge in adulthood might thus be resulting from the priming effects of an early-life infection on the population of adult hippocampal microglia. This may lead to an exaggerated pro-inflammatory response with detrimental effects on adult hippocampal neurogenesis and cognition.

Neuroimmune system activation is not solely induced by peripheral bacterial components. The consequences of direct modulation of central cytokines and/or central induction of innate immune cells on the hippocampus are moderately studied. An example is TNFα injection at P3 and P5, increasing anxiety-like behaviors in male mice (Babri et al., 2014b). However, other cytokine overexpression levels have not been elegantly studied. For some years now, viral infections have been considered a contributing factor to the development of neuropsychological disorders, including hippocampal related dysfunction (Das and Basu, 2011). Various viruses are used to investigate disruption in hippocampal development and adult neurogenesis, e.g., the lymphocytic choriomengitis virus (LCMV) (Pearce et al., 1996; Sharma et al., 2002; Orr et al., 2010), Borna disease virus (BDV; Zocher et al., 2000; Sauder et al., 2001) or polyinosinic:polycytidylic acid (Poly I:C; Galic et al., 2009). Each of these viruses induces different phenotypical changes. Poly I:C intracerebroventricular injection at P14 induces short time elevations of pro-inflammatory cytokine IL-1β in the hippocampus and adult-onset deficits in contextual fear conditioning (Galic et al., 2009). But earlier central administration of LCMV at P4 produces lasting IL-1β induction with subsequent loss of cells in the granular cell layer (Sharma et al., 2002; Orr et al., 2010) and reduced levels of progenitor cells in the DG (Sharma et al., 2002), without affecting other hippocampal regions (Pearce et al., 1996). This virus induced phenotype could be reduced by the use of an anti-inflammatory agent to block IL-1β, which restored the granular cell numbers in adulthood (Orr et al., 2010). The BDV virus typically induces apoptosis of DG cells at 27 and 33 days post infection, possibly mediated by a reduction in neurotrophins in this brain region (Zocher et al., 2000) and further impairs MWM performance in adulthood, correlating to chemokine expression levels (Sauder et al., 2001). How these virus infections early in life mechanistically affect the hippocampus is unfortunately poorly understood to date (Das and Basu, 2011).

Altogether, early-life peripheral infection immediately increases pro-inflammatory cytokines in the hippocampus and exerts lasting effects on hippocampal structure, but evokes only subtle alterations in hippocampal neurogenesis and functionality under basal condition. After exposure to a second immunological challenge in adulthood, however, a history of early-life infection has aversive effects on cognitive function related to an exaggerated pro-inflammatory response in the hippocampus. On the other hand, viral infection that induces a central stimulation of the immune system leaves detrimental effects on the DG, affecting adult hippocampal neurogenesis and cognitive functions. The lasting effect of early-life infection on hippocampal microglia suggests that a programming effect of peripheral and ultimately central immune system activity plays an important role in the lasting effects of hippocampal structure and cognitive functions.

## THE INTERPLAY OF THE DIFFERENT ELEMENTS IN THE EARLY-LIFE ENVIRONMENT

**FIGURE 1:**
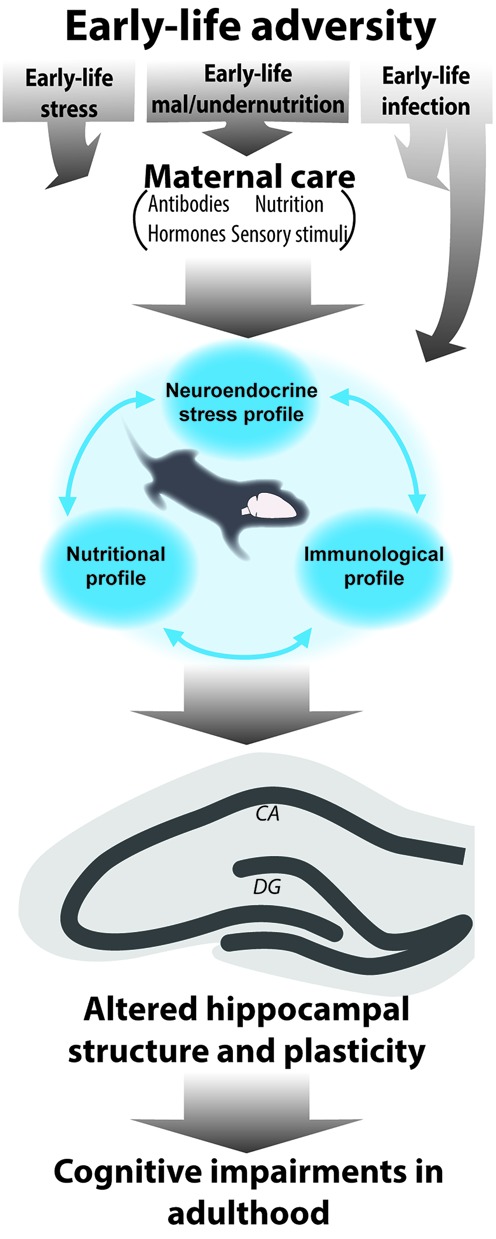
**Schematic representation of the interrelated role of different early-life elements for the consequences of early-life adversity.** Early-life adversities in the form of early-life stress, under/malnutrition and infection are known to modulate hippocampal development and altogether determine hippocampal structure and function in adulthood with adverse effects on learning and memory. During the early sensitive period of development the offspring is fully dependent on the mother. Maternal care encompasses several elements (sensory stimuli, transfer of nutrition, hormones, and antibodies). In fact it is mostly via disruption of maternal care (with exception of early-life infection which can directly act upon the offspring) that early adversities will elicit disruptions in hormonal, (neuro)inflammatory and nutritional profiles in the offspring. Because these elements affect one another, they will ultimately act synergistically to modulate hippocampal structure and function throughout life.

The discussed consequences of early-life stress, nutrition, and immune activation can all be considered forms of early-life adversity. Although limited studies have examined the integrated role of these elements, the presented evidence in the above sections clearly points to the fact that challenges, even when very different in nature (disruption of maternal care, malnutrition, or immune), lead to strikingly similar outcomes of disrupted hippocampal structure and plasticity later in life as well as cognitive impairments. Knowing that these systems are tightly related and that they affect each other, it is reasonable to assume that the current models of early-life stress, malnutrition and infections discussed up to now elicit effects on all these different levels (**Figure 1**) and that it is the synergistic effects of all of these components that lead to the observed outcome rather than only the experimentally modulated one. In the upcoming section, we will discuss the current evidence and missing links for this hypothesis. Because the tight interaction and possible synergistic effects of stress and nutrition on neurocognitive development has been recently reviewed and discussed both prenatally (Monk et al., 2013) as well as postnatally (Lucassen et al., 2013), we will here focus on the interaction of early-life stress and malnutrition with the immune activation.

### WHAT IS THE EVIDENCE FOR AN INTERACTION BETWEEN EARLY-LIFE IMMUNE ACTIVATION AND EARLY-LIFE ADVERSITY?

Early-life adversities like stress and malnutrition not only lead to the previously described effects on cognitive and hippocampal function but also to changes in adult immunological function. The stress and immune systems have a strong interactive profile, illustrated by, e.g., the immune-suppressive effect of corticosteroids (Tsigos and Chrousos, 2002; Chaplin, 2010). Evidence of this relation in early-life has been provided by the enhanced pro-inflammatory status, both basally as well as in response to stress (Carpenter et al., 2010; Chen et al., 2010), of adult individuals with a history of early-life adversity, such as children raised in poor socioeconomic status households or who suffered from childhood maltreatment. This association has been confirmed by preclinical studies as well. In general, early-life stress paradigms (pre- and postnatal) lead to an immediate immunosuppressive state, e.g., a reduced lymphoproliferative response of the thymus (Llorente et al., 2007), downregulation of anti-inflammatory IL-10 and pro-inflammatory IL-1β (Dimatelis et al., 2012) and reduction of lipobinding protein (LBP), that regulates innate immune system pathogen presentation by microglia cells (Wei et al., 2012). These alterations in the immunoreactive profile in early-life are further accompanied by central changes in microglia morphology and number of Iba1 (Diz-Chaves et al., 2012) or lectin immunoreactive cells (Gómez-González and Escobar, 2009b).

In adulthood, the programming effect of early-life adversity by maternal deprivation on immune regulation is further illustrated by enhanced IL-1β responsiveness due to elevated IL-1 receptor levels at the post-synapse of adult hippocampal neurons (Viviani et al., 2013). In addition, the inflammatory response to an inflammatory challenge (systemic LPS injection) in the hippocampus of prenatally stressed mice is exaggerated in the reactivity of microglia and expression of pro-inflammatory cytokines (Diz-Chaves et al., 2013). Altogether, early-life stress tends to have a programming effect on neuroimmune functions, mainly resulting in an immediate immunosuppressive, but pro-inflammatory state in adulthood, which triggers an exaggerated neuroimmune response defined by cytokine secretion and microglia activity upon an immune challenge. How the early-life adversity-induced pro-inflammatory adult profile in the brain interacts with the other changes in brain structure and how these altogether lead to the observed cognitive impairments needs to be further investigated.

Next to the evident programming effects of early-life stress on neuroimmune functions, possible lasting effects of inflammatory challenges during early-life on HPA axis activity need to be considered as well. Early-life infection generally leads to a direct elevation of circulating glucocorticoids in early-life (Bilbo et al., 2005a) and while basal CORT was not affect by early-life infection at P14, the level of phosphorylated GR is significantly higher in the prefrontal cortex, but not the hippocampus (Dinel et al., 2014). In contrast, early infection does not affect basal and/or stress-induced CORT in a lasting manner (Bilbo et al., 2006; Walker et al., 2010; Babri et al., 2014a; Dinel et al., 2014). In line with this, CORT secretion following adult LPS exposure seems independent of the early-life history of exposure to stress or infection (Bilbo et al., 2005a, 2007; Kohman et al., 2008). However there is evidence indicating that early-life infected animals exhibit prolonged CORT elevations accompanied by a greater content of pro-inflammatory IL-1β and TNFα in the hippocampus upon adult stress exposure (Walker et al., 2010) while exposure to high doses of the pro-inflammatory cytokine TNFα increased stress-induced CORT release (Babri et al., 2014b).

Clearly, in the activation of neuroimmune cells induced either by a peripheral immune challenge or by early-life adversity, the BBB plays a pivotal role. There is evidence that development of the BBB is hampered after exposure to perinatal stress and exposure to an early-life immune challenge, revealing elevated BBB leakage in among other areas the hippocampus (Gómez-González and Escobar, 2009a). Whether these changes in early-life stress induced BBB leakage are related to changes in neuroimmune functioning after early-life stress remains to be determined.

### WHAT IS THE EVIDENCE FOR AN INTERACTION BETWEEN EARLY-LIFE MALNUTRITION AND NEUROIMMUNE ACTIVATION?

Next to early-life stress, also early-life nutritional insults can affect the neuroimmune system. For example, there are indications for a strong association between circulating leptin levels and the suppression of lymphoproliferative responses and pro-inflammatory cytokine secretion in protein malnourished infants, both before and after recovery following refeeding (Palacio et al., 2002).

Similar indications are provided by preclinical studies where adult offspring of food-restriction dams have increased basal immune activity (measured as C-reactive protein) in female offspring at 9 months of age, but reduced cytokine induction (IL-1β and IL-6 secretion) in response to a second immune insult with LPS (Desai et al., 2009). Similarly, adult offspring of dams that were protein-deprived during lactation show an impaired responsiveness to a peripheral immune challenge, that was accompanied by elevated levels of basal and response CORT (Barja-Fidalgo et al., 2003). Lipid content of the diet early in life seems to be a strong modulator of neuroimmune functioning throughout life. Indeed, offspring of dams fed high-fat and high-trans fat during pregnancy and lactation exhibit increased basal immune activity (C-reactive protein) at birth and increased active microglia in adult (Bilbo and Tsang, 2010) associated with improved performance in the MWM. These basal changes are accompanied by an exaggerated peripheral and hippocampal IL-1β response to adult LPS (Bilbo and Tsang, 2010), classically known to activate microglia (Van Dam et al., 1992). Interestingly, when maternally deprived rats (P9) are weaned on a high fat diet during adult life, they present an increased pro-inflammatory modulation of IL-1β and TNFα in the hypothalamus (Mela et al., 2012). Omega-3 FAs in particular appear responsible for these observations as they activate neuroprotective signaling pathways (Calon and Cole, 2007) and act upon immune regulators, by, e.g., blockage of the NFκB signaling pathway (Singer et al., 2008). In fact, male offspring of omega-3 deficient dams exhibit a promotion of reactive inflammatory microglia and elevated pro-inflammatory cytokines in the hippocampus at P21 (Madore et al., 2014). It remains elusive how the changes in dietary fat composition early in life and subsequent priming of microglia further relate to levels of adult hippocampal neurogenesis.

One of the possible mediators of the interaction between nutritional intake and immune system is leptin, which is secreted by white adipose tissue (Fernández-Riejos et al., 2010). In fact, rats treated with LPS at P10 have increased food intake in adulthood associated with elevated circulating leptin levels. A second immune challenge (LPS at 7–8 weeks of age), while leading to an elevation in leptin serum level in animals that were never exposed to infection before, did not alter leptin levels in the neonatally infected animals (Iwasa et al., 2010). Moreover, neonatal overfeeding, similarly to the combination of early-life stress and a high fat diet as discussed above (Mela et al., 2012), leads to microgliosis in the hypothalamic regions, including the PVN of the hypothalamus, a key nucleus in the regulation of HPA axis activity that can be triggered by interleukin-1 as well (Berkenbosch et al., 1987). Especially in this region, microglia activation is overly exaggerate upon an immune challenge with LPS in adulthood (Ziko et al., 2014). Interestingly, these manipulations lead to a disruption in the patterns of leptin, coinciding with the leptin surge for normal hypothalamic development (Ahima et al., 1998; Ahima and Hileman, 2000). Thus, a disrupted pattern of leptin secretion and induced (neuro) inflammation by these manipulations may play an important role in programming of the cognitive functions (Miller and Spencer, 2014). It is remarkable that entirely different manipulations as prenatal stress (Diz-Chaves et al., 2013), being raised by a dam exposed to high fat diet during pregnancy and lactation (Bilbo and Tsang, 2010) and neonatal overfeeding (Ziko et al., 2014) reset the neuroimmune function and lead to exaggerated microgliosis in response to a subsequent immune challenge in adulthood.

Although not extensively studied during early-life, current evidence on the dietary supply of methyl donors modulating the present levels of homocysteine in the (developing) brain (Blaise et al., 2007; Troen et al., 2008), with a deficiency leading to hyperhomocysteinemia, further suggests a role for dietary methyl donors in microglia properties and activity in adulthood. Hyperhomocysteinemia is associated with an elevated levels of homocystein-presenting apoptotic cells, and also with enhanced proliferation of microglia in the brain (Zou et al., 2010). Moreover, hyper-homocysteinemia can be a risk factor or marker of neurodegenerative disorders in which cognitive dysfunction and neuroimmune functioning play an important role (Morris, 2003; Van Dam and Van Gool, 2009). However, to date, the exact relation of early-life methyl donor supply and neuroimmune functions remains elusive.

Exciting new evidence further supports a strong interaction between nutrition and immune system in the programming of hippocampal structure and function. A recent paper by Liu et al. (2014) proposed a pathway of “lactocrine” programming of hippocampal development and function by maternal deficiency of TNFα, resulting in altered chemokine composition of the mother. In fact, TNFα deficiency in mothers milk lead to impaired hippocampal proliferation and spatial memory in the offspring of these animals, a clear indication of programming via nutritionally provided immune effector messengers (Liu et al., 2014). The exact pathway of TNFα and the role of chemokines that lead to alterations in hippocampal development, adult learning and memory, remains to be explored (Parylak et al., 2014).

Altogether, dietary composition during critical sensitive periods of development seem to be strongly involved in the immediate and lasting effects on the innate immune system, with a tendency to an immediate immunosuppressive response being associated with protein and fat malnourishment, and an enhanced pro-inflammatory activity induced by high fat diets associated with an sub-optimal neuroimmune response (too little or exaggerated) in response to later life immune challenges. How the early-life nutrition directly programs neuroimmune function and interacts with the neuroendocrine system, and programs cognitive functions and hippocampal neurogenesis in later life requires further study.

In the previous section we have highlighted some of the key elements of the early-life environment that might play an important role in the programming of cognitive functions by early-life adversity. As evident from the studies that we discussed these elements clearly do not act alone but rather in a synergistic manner. We discussed some of the possible mechanisms that could mediate the effects of early-life stress, malnutrition, and infection and discussed the evidence for their interactive profile. However clearly our discussion is not exhaustive and other equally important paths and mediators responsible for the final programming effect could be considered. For example, next to leptin, ghrelin a pancreatic hormone released upon hunger can influence not only eating behavior but stress, immune function as well as cognition (Diz-Chaves, 2011). Clearly having to consider so many different elements simultaneously renders the picture very complex and questions which are the best systems to target to prevent and/or reverse the deleterious effects of early-life adversity. In the following section, we will discuss some of the intervention strategies that have been explored up to date.

## EARLY-LIFE ADVERSITY; OPPORTUNITIES FOR INTERVENTION LATER IN LIFE

Adversities in the early-life period provoke thus immediate and programmed effects on different levels with lasting consequences for hippocampal function. Identification of these consequences and the different systems at play during early-life is essential to design optimal intervention studies to counteract the more complete set of consequences following early-life adversity. In recent years, multiple intervention studies have been performed to counteract either the lack of nutritional components, the consequences of early-life stress or the pro-inflammatory state after early-life infections. For instance, clinical research revealed the potential of high levels of maternal warmth (regarded as a positive experience) to overcome the programmed effects of the aversive low socioeconomic status on the immune system during early-life (Chen et al., 2010). However, considering the evidence presented in this review that these systems interact and affect each other so tightly and that they might thus act synergistically to program brain structure and function for life, the question arises as to which consequences of early-life adversity to target and whether there is a crucial time window for these interventions for optimal beneficial effects of these interventions. Here, we will discuss a few examples of potential intervention studies.

Because changes of HPA axis modulators are suggested as potential regulators of the lasting changes following early-life stress, suppression of these modulators has been investigated as a possible intervention in later life. For example, selective blockage of CRF receptor 1 immediately after the first week after chronic early-life stress exposure from postnatal day 10–17 in rats prevents hippocampal impairments in cognitive functioning and long-term potentiation (Ivy et al., 2010).

Enriching the later life environment, a manipulation that is known to stimulate hippocampal neurogenesis and improve performance of hippocampus related spatial behavioral tasks in adulthood (Kempermann et al., 1997; Nilsson et al., 1999), has been explored as well. For example, housing maternally separated rats in an enriched environmental condition during adulthood reversed the early-life stress induced changes in hippocampal GR and CRF expression (Francis et al., 2002). These manipulations do not only modify HPA axis activity but also affect neuroimmune functioning and activity of glial cells (Olah et al., 2009; Williamson et al., 2012; Gebara et al., 2013). In addition, there is evidence that enriching early-life environment by artificially increasing sensory stimuli by the mother (via handling) interferes with the adult pro-inflammatory programming of early-life *E. coli* infection (Bilbo et al., 2007). The adult LPS-induced increase of microglia (CD11b) and astrocyte (GFAP) markers and IL-1β levels in the blood and different brain regions of animals with a history of *E. coli* exposure was fully prevented by early-life handling. These data clearly suggest a strong interaction between sensory stimuli and infection early in life in the programming of the adult neuroimmune system (Bilbo et al., 2007).

A final manner to intervene with the consequences of early-life stress is modulation at the level of the epigenome. Early-life stress and early-life nutrition program later life function through alterations in chromatin structure and gene expression as became evident from clinical and animal studies. There is indeed increasing evidence that epigenetic mechanisms might be responsible for the early-life adversity induced life-long alterations in gene expression. (Heijmans et al., 2008; Murgatroyd et al., 2009; Steegers-Theunissen et al., 2009; Canani et al., 2011; Chen et al., 2012; Lucassen et al., 2013). Moreover, epigenetics mechanisms play a role in neuroinflammatory responses as well (Garden, 2013). Which are the factors regulating epigenetic mechanisms is yet unclear, however, there is growing interest in the role that nutrition might play in this context (Lucassen et al., 2013; Spencer, 2013). Nutritional interventions to prevent or reverse these epigenetic alterations have been only explored concerning metabolic programming but might certainly have the potential to intervene with the deleterious programming by early-life adversity of brain structure and function (Lucassen et al., 2013). For instance, folic acid supplementation to the offspring of protein-restricted diet fed dams during adolescence altered the protein-restricted metabolic outcome and modified the epigenetic alterations (Burdge et al., 2009). In addition, folate deficiency of the maternal diet during gestation negatively influences hippocampal developmental neurogenesis, but supplementation with the interrelated methyl donor choline modified some of these effects on the neural progenitor cells (Craciunescu et al., 2010). But can dietary intervention later in life prevent or reverse the early-life adversity induced phenotype? Weaver et al. (2005) provided evidence that the programming effect of maternal care during early-life on the epigenetic modifications of the GR remain sensitive to alterations in adulthood, as central infusion with L-methionine could reverse the programmed effects of maternal care.

Overall, these studies indicate that environmental, nutritional, and pharmacological interventions, either during early-life or in adulthood, have the potential to modulate one or more consequences of early-life adversity. Currently, intervention studies lack some depth on the interplay of stress mediators, neuroimmune activity and the nutritional profile in how they might synergistically modulate hippocampal structure and function. Addressing the complexity of the early-life environment at large, rather than focusing on a single element will provide the necessary information to design new interventions, or a combination of interventions, that may fully prevent and/or reverse the consequences of early-life adversity.

## FINAL CONCLUSION

Well-known factors such as genetic vulnerability, gender, life style, and aging contribute to disorder vulnerability. In addition, early-life adversity further determines brain susceptibility to develop adult-onset psychopathologies and cognitive impairments later in life. Multiple elements (including stress, nutrition, and infections) in the early-life environment are crucial for proper hippocampal development, and structure and function in adulthood. Thus there is growing evidence that disruption of either of these elements has detrimental effects on cognitive functions, hippocampal structure, neurogenesis and the activity of neuroimmune cells in the hippocampus. Here, we have focused on how these different elements might interplay during early-life adversity and elicit similar effects on hippocampal neurogenesis and cognition in adulthood. Even though the interplay of these three elements is generally not considered in depth, the ultimate consequences are probably a synergistic effect and combination of these elements. Considering the intense cross talk between these elements and how they, together, program hippocampal structure and function, will provide important insights and contribute to novel targets for pharmacological, nutritional or life style interventions after early-life adversity.

## Conflict of Interest Statement

The authors declare that the research was conducted in the absence of any commercial or financial relationships that could be construed as a potential conflict of interest.

## References

[B1] Ahima R. S. Hileman S. M. (2000). Postnatal regulation of hypothalamic neuropeptide expression by leptin: implications for energy balance and body weight regulation. *Regul. Pept.* 92 1–7 10.1016/S0167-0115(00)00142-7 11024558

[B2] Ahima R. S. Prabakaran D. Flier J. S. (1998). Postnatal leptin surge and regulation of circadian rhythm of leptin by feeding. Implications for energy homeostasis and neuroendocrine function. *J. Clin. Invest.* 101 1020–1027 10.1172/JCI1176 9486972 PMC508653

[B3] Ahmad A. Moriguchi T. Salem N. (2002). Decrease in neuron size in docosahexaenoic acid-deficient brain. *Pediatr. Neurol.* 26 210–218 10.1016/S0887-8994(01)00383-6 11955929

[B4] Aisa B. Tordera R. Lasheras B. Del Rio J. Ramirez M. J. (2007). Cognitive impairment associated to HPA axis hyperactivity after maternal separation in rats. *Psychoneuroendocrinology* 32 256–266 10.1016/j.psyneuen.2006.12.013 17307298

[B5] Allen L. H. (2012). B Vitamins in breast milk: relative importance of maternal status and intake, and effects on infant status and function. *Adv. Nutr.* 3 362–369 10.3945/an.111.001172 22585913 PMC3649471

[B6] Allen N. J. Barres B. A. (2009). Glia – more than just brain glue. *Nature* 457 675–677 10.1038/457675a 19194443

[B7] Altman J. Bayer S. A. (1990). Migration and distribution of two populations of hippocampal granule cell precursors during the perinatal and postnatal periods. *J. Comp. Neurol.* 301 365–381 10.1002/cne.903010304 2262596

[B8] André C. Dinel A. Ferreira G. Layé S. Castanon N. (2014). Diet-induced obesity progressively alters cognition, anxiety-like behavior and lipopolysaccharide-induced depressive-like behavior: focus on brain indoleamine 2,3-dioxygenase activation. *Brain Behav. Immun.* 41 10–21 10.1016/j.bbi.2014.03.012 24681251

[B9] Arnold S. E. Trojanowski J. Q. (1996). Human fetal hippocampal development: I. Cytoarchitecture, myeloarchitecture, and neuronal morphologic features. *J. Comp. Neurol.* 367 274–292 10.1002/(SICI)1096-9861(19960401)367:2<274::AID-CNE9>3.0.CO;2-2 8708010

[B10] Babri S. Doosti M.-H. Salari A. (2014a). Strain-dependent effects of prenatal maternal immune activation on anxiety- and depression-like behaviors in offspring. *Brain Behav. Immun.* 37 164–176 10.1016/j.bbi.2013.12.003 24326014

[B11] Babri S. Doosti M.-H. Salari A. (2014b). Tumor necrosis factor-alpha during neonatal brain development affects anxiety- and depression-related behaviors in adult male and female mice. *Behav. Brain Res.* 261 305–314 10.1016/j.bbr.2013.12.037 24398264

[B12] Bagot R. C. van Hasselt F. N. Champagne D. L. Meaney M. J. Krugers H. J. Joëls M. (2009). Maternal care determines rapid effects of stress mediators on synaptic plasticity in adult rat hippocampal dentate gyrus. *Neurobiol. Learn. Mem.* 92 292–300 10.1016/j.nlm.2009.03.004 19292996

[B13] Bale T. L. Baram T. Z. Brown A. S. Goldstein J. M. Insel T. R. McCarthy M. M. (2010). Early life programming and neurodevelopmental disorders. *Biol. Psychiatry* 68 314–319 10.1016/j.biopsych.2010.05.028 20674602 PMC3168778

[B14] Banks W. A. Plotkin S. R. Kastin A. J. (1995). Permeability of the blood-brain barrier to soluble cytokine receptors. *Neuroimmunomodulation* 2 161–165 10.1159/000096887 8646566

[B15] Barja-Fidalgo C. Souza E. P. G. Silva S. V. Rodrigues A. L. Anjos-Valotta E. A. Sannomyia P. (2003). Impairment of inflammatory response in adult rats submitted to maternal undernutrition during early lactation: role of insulin and glucocorticoid. *Inflamm. Res.* 52 470–476 10.1007/s00011-003-1207-3 14652681

[B16] Barker D. (2004). Developmental origins of adult health and disease. *J. Epidemiol. Community Health* 58 114–115 10.1136/jech.58.2.114 14729887 PMC1732687

[B17] Barker D. Barker M. Fleming T. Lampl M. (2013). Developmental biology: support mothers to secure future public health. *Nature* 504 209–211 10.1038/504209a 24350368

[B18] Bedi K. S. (1991). Early-life undernutrition causes deficits in rat dentate gyrus granule cell number. *Experientia* 47 1073–1074 10.1007/BF01923346 1936206

[B19] Bedi K. S. (1992). Spatial-learning ability of rats undernourished during early postnatal life. *Physiol. Behav.* 51 1001–1007 10.1016/0031-9384(92)90084-F 1615037

[B20] Beltz B. S. Tlusty M. F. Benton J. L. Sandernan D. C. (2007). Omega-3 fatty acids upregulate adult neurogenesis. *Neurosci. Lett.* 415 154–158 10.1016/j.neulet.2007.01.010 17240063 PMC1892224

[B21] Benton D. (2010). The influence of dietary status on the cognitive performance of children. *Mol. Nutr. Food Res.* 54 457–470 10.1002/mnfr.200900158 20077417

[B22] Berkenbosch F. van Oers J. del Rey A. Tilders F. Besedovsky H. (1987). Corticotropin-releasing factor-producing neurons in the rat activated by interleukin-1. *Science* 238 524–526 10.1126/science.2443979 2443979

[B23] Bertrand P. C. O’Kusky J. R. Innis S. M. (2006). Maternal dietary (n-3) fatty acid deficiency alters neurogenesis in the embryonic rat brain. *J. Nutr.* 136 1570–1575. 16702323 10.1093/jn/136.6.1570

[B24] Bilbo S. D. Barrientos R. M. Eads A. S. Northcutt A. Watkins L. R. Rudy J. W. (2008). Early-life infection leads to altered BDNF and IL-1beta mRNA expression in rat hippocampus following learning in adulthood. *Brain Behav. Immun.* 22 451–455 10.1016/j.bbi.2007.10.003 17997277

[B25] Bilbo S. D. Biedenkapp J. C. Der-Avakian A. Watkins L. R. Rudy J. W. Maier S. F. (2005a). Neonatal infection-induced memory impairment after lipopolysaccharide in adulthood is prevented via caspase-1 inhibition. *J. Neurosci.* 25 8000–8009 10.1523/JNEUROSCI.1748-05.2005 16135757 PMC6725459

[B26] Bilbo S. D. Levkoff L. H. Mahoney J. H. Watkins L. R. Rudy J. W. Maier S. F. (2005b). Neonatal infection induces memory impairments following an immune challenge in adulthood. *Behav. Neurosci.* 119 293–301 10.1037/0735-7044.119.1.293 15727533

[B27] Bilbo S. D. Newsum N. J. Sprunger D. B. Watkins L. R. Rudy J. W. Maier S. F. (2007). Differential effects of neonatal handling on early life infection-induced alterations in cognition in adulthood. *Brain Behav. Immun.* 21 332–342 10.1016/j.bbi.2006.10.005 17126527

[B28] Bilbo S. D. Rudy J. W. Watkins L. R. Maier S. F. (2006). A behavioural characterization of neonatal infection-facilitated memory impairment in adult rats. *Behav. Brain Res.* 169 39–47 10.1016/j.bbr.2005.12.002 16413067

[B29] Bilbo S. D. Schwarz J. M. (2009). Early-life programming of later-life brain and behavior: a critical role for the immune system. *Front. Behav. Neurosci.* 3:9 10.3389/neuro.08.014.2009 PMC2737431 19738918

[B30] Bilbo S. D. Tsang V. (2010). Enduring consequences of maternal obesity for brain inflammation and behavior of offspring. *FASEB J.* 24 2104–2115 10.1096/fj.09-144014 20124437

[B31] Blaise S. A. Nédélec E. Schroeder H. Alberto J.-M. Bossenmeyer-Pourié C. Guéant J.-L. (2007). Gestational vitamin B deficiency leads to homocysteine-associated brain apoptosis and alters neurobehavioral development in rats. *Am. J. Pathol.* 170 667–679 10.2353/ajpath.2007.060339 17255334 PMC1851855

[B32] Bland S. T. Beckley J. T. Watkins L. R. Maier S. F. Bilbo S. D. (2010a). Neonatal *Escherichia coli* infection alters glial, cytokine, and neuronal gene expression in response to acute amphetamine in adolescent rats. *Neurosci. Lett.* 474 52–57 10.1016/j.neulet.2010.03.006 20223277 PMC2865898

[B33] Bland S. T. Beckley J. T. Young S. Tsang V. Watkins L. R. Maier S. F. (2010b). Enduring consequences of early-life infection on glial and neural cell genesis within cognitive regions of the brain. *Brain Behav. Immun.* 24 329–338 10.1016/j.bbi.2009.09.012 19782746 PMC2826544

[B34] Bredy T. W. Grant R. J. Champagne D. L. (2003). Maternal care influences neuronal survival in the hippocampus of the rat. *Eur. J. Neurosci.* 18 2903–2909 10.1046/j.1460-9568.2003.02965.x 14656341

[B35] Brown A. S. (2006). Prenatal infection as a risk factor for schizophrenia. *Schizophr. Bull.* 32 200–202 10.1093/schbul/sbj052 16469941 PMC2632220

[B36] Brown A. S. van Os J. Driessens C. Hoek H. W. Susser E. S. (2000). Further evidence of relation between prenatal famine and major affective disorder. *Am. J. Psychiatry* 157 190–195 10.1176/appi.ajp.157.2.190 10671386

[B37] Brown C. P. Smotherman W. P. Levine S. (1977). Interaction-induced reduction in differential maternal responsiveness: an effect of cue-reduction or behavior? *Dev. Psychobiol.* 10 273–280 10.1002/dev.420100311 863123

[B38] Brunson K. L. (2005). Mechanisms of late-onset cognitive decline after early-life stress. *J. Neurosci.* 25 9328–9338 10.1523/JNEUROSCI.2281-05.2005 16221841 PMC3100717

[B39] Brunson K. L. Baram T. Z. Bender R. A. (2005). Hippocampal neurogenesis is not enhanced by lifelong reduction of glucocorticoid levels. *Hippocampus* 15 491–501 10.1002/hipo.20074 15744738 PMC2921196

[B40] Burdge G. C. Lillycrop K. A. Phillips E. S. Slater-Jefferies J. L. Jackson A. A. Hanson M. A. (2009). Folic acid supplementation during the juvenile-pubertal period in rats modifies the phenotype and epigenotype induced by prenatal nutrition. *J. Nutr.* 139 1054–1060 10.3945/jn.109.104653 19339705

[B41] Calon F. Cole G. (2007). Neuroprotective action of omega-3 polyunsaturated fatty acids against neurodegenerative diseases: evidence from animal studies. *Prostaglandins Leukot. Essent. Fatty Acids* 77 287–293 10.1016/j.plefa.2007.10.019 18037281

[B42] Cameron H. A. Gould E. (1994). Adult neurogenesis is regulated by adrenal steroids in the dentate gyrus. *Neuroscience* 61 203–209 10.1016/0306-4522(94)90224-0 7969902

[B43] Campbell L. F. Bedi K. S. (1989). The effects of undernutrition during early life on spatial learning. *Physiol. Behav.* 45 883–890 10.1016/0031-9384(89)90210-2 2780873

[B44] Canani R. B. Costanzo M. D. Leone L. Bedogni G. Brambilla P. Cianfarani S. (2011). Epigenetic mechanisms elicited by nutrition in early life. *Nutr. Res. Rev.* 24 198–205 10.1017/S0954422411000102 22008232

[B45] Capuron L. Miller A. H. (2011). Immune system to brain signaling: neuropsychopharmacological implications. *Pharmacol. Ther.* 130 226–238 10.1016/j.pharmthera.2011.01.014 21334376 PMC3072299

[B46] Carpenter L. L. Gawuga C. E. Tyrka A. R. Lee J. K. Anderson G. M. Price L. H. (2010). Association between plasma IL-6 response to acute stress and early-life adversity in healthy adults. *Neuropsychopharmacology* 35 2617–2623 10.1038/npp.2010.159 20881945 PMC2978751

[B47] Carrié I. Guesnet P. Bourre J. M. Francès H. (2000). Diets containing long-chain n-3 polyunsaturated fatty acids affect behaviour differently during development than ageing in mice. *Br. J. Nutr.* 83 439–447 10.1017/S0007114500000544 10858702

[B48] Cartier L. Hartley O. Dubois-Dauphin M. Krause K.-H. (2005). Chemokine receptors in the central nervous system: role in brain inflammation and neurodegenerative diseases. *Brain Res. Brain Res. Rev.* 48 16–42 10.1016/j.brainresrev.2004.07.021 15708626

[B49] Castro C. A. Tracy M. Rudy J. W. (1989). Early-life undernutrition impairs the development of the learning and short-term memory processes mediating performance in a conditional-spatial discrimination task. *Behav. Brain Res.* 32 255–264 10.1016/S0166-4328(89)80058-0 2496701

[B50] Chaboub L. S. Deneen B. (2013). Astrocyte form and function in the developing central nervous system. *Semin. Pediatr. Neurol.* 20 230–235 10.1016/j.spen.2013.10.003 24365570 PMC3874818

[B51] Champagne D. L. Bagot R. C. van Hasselt F. Ramakers G. Meaney M. J. de Kloet E. R. (2008). Maternal care and hippocampal plasticity: evidence for experience-dependent structural plasticity, altered synaptic functioning, and differential responsiveness to glucocorticoids and stress. *J. Neurosci.* 28 6037–6045 10.1523/JNEUROSCI.0526-08.2008 18524909 PMC6670331

[B52] Chaplin D. D. (2003). 1. Overview of the immune response. *J. Allergy Clin. Immunol.* 111 S442–S459 10.1067/mai.2003.125 12592292

[B53] Chaplin D. D. (2010). Overview of the immune response. *J. Allergy Clin. Immunol.* 125 S3–S23 10.1016/j.jaci.2009.12.980 20176265 PMC2923430

[B54] Chen E. Miller G. E. Kobor M. S. Cole S. W. (2010). Maternal warmth buffers the effects of low early-life socioeconomic status on pro-inflammatory signaling in adulthood. *Mol. Psychiatry* 16 729–737 10.1038/mp.2010.53 20479762 PMC2925055

[B55] Chen J. Evans A. N. Liu Y. Honda M. Saavedra J. M. Aguilera G. (2012). Maternal deprivation in rats is associated with corticotrophin-releasing hormone (CRH) promoter hypomethylation and enhances CRH transcriptional responses to stress in adulthood. *J. Neuroendocrinol.* 24 1055–1064 10.1111/j.1365-2826.2012.02306.x 22375940 PMC3380160

[B56] Chugani H. T. Behen M. E. Muzik O. Juhász C. Nagy F. Chugani D. C. (2001). Local brain functional activity following early deprivation: a study of postinstitutionalized Romanian orphans. *Neuroimage* 14 1290–1301 10.1006/nimg.2001.0917 11707085

[B57] Cignacco E. Hamers J. P. H. Stoffel L. van Lingen R. A. Gessler P. McDougall J. (2007). The efficacy of non-pharmacological interventions in the management of procedural pain in preterm and term neonates. A systematic literature review. *Eur. J. Pain* 11 139–152 10.1016/j.ejpain.2006.02.010 16580851

[B58] Coelho R. Viola T. W. Walss-Bass C. Brietzke E. Grassi-Oliveira R. (2014). Childhood maltreatment and inflammatory markers: a systematic review. *Acta Psychiatr. Scand.* 129 180–192 10.1111/acps.12217 24205846

[B59] Cope E. C. Gould E. (2013). Cytokines make an indelible impression on neural stem cells. *Cell Stem Cell* 13 507–508 10.1016/j.stem.2013.10.011 24209754

[B60] Costello E. J. Worthman C. Erkanli A. Angold A. (2007). Prediction from low birth weight to female adolescent depression: a test of competing hypotheses. *Arch. Gen. Psychiatry* 64 338–344 10.1001/archpsyc.64.3.338 17339522

[B61] Coupé B. Dutriez-Casteloot I. Breton C. Lefèvre F. Mairesse J. Dickes-Coopman A. (2009). Perinatal undernutrition modifies cell proliferation and brain-derived neurotrophic factor levels during critical time-windows for hypothalamic and hippocampal development in the male rat. *J. Neuroendocrinol.* 21 40–48 10.1111/j.1365-2826.2008.01806.x 19094092

[B62] Craciunescu C. N. Johnson A. R. Zeisel S. H. (2010). Dietary choline reverses some, but not all, effects of folate deficiency on neurogenesis and apoptosis in fetal mouse brain. *J. Nutr.* 140 1162–1166 10.3945/jn.110.122044 20392884 PMC2869500

[B63] Cunningham C. L. Martínez-Cerdeño V. Noctor S. C. (2013). Microglia regulate the number of neural precursor cells in the developing cerebral cortex. *J. Neurosci.* 33 4216–4233 10.1523/JNEUROSCI.3441-12.2013 23467340 PMC3711552

[B64] Dammann O. Kuban K. C. K. Leviton A. (2002). Perinatal infection, fetal inflammatory response, white matter damage, and cognitive limitations in children born preterm. *Ment. Retard. Dev. Disabil. Res. Rev.* 8 46–50 10.1002/mrdd.10005 11921386

[B65] Danese A. Moffitt T. E. Harrington H. Milne B. J. Polanczyk G. Pariante C. M. (2009). Adverse childhood experiences and adult risk factors for age-related disease: depression, inflammation, and clustering of metabolic risk markers. *Arch. Pediatr. Adolesc. Med.* 163 1135–1143 10.1001/archpediatrics.2009.214 19996051 PMC3560401

[B66] Dangat K. D. Mehendale S. S. Yadav H. R. Kilari A. S. Kulkarni A. V. Taralekar V. S. (2010). Long-chain polyunsaturated fatty acid composition of breast milk in pre-eclamptic mothers. *Neonatology* 97 190–194 10.1159/000252971 19864925

[B67] Das S. Basu A. (2008). Inflammation: a new candidate in modulating adult neurogenesis. *J. Neurosci. Res.* 86 1199–1208 10.1002/jnr.21585 18058947

[B68] Das S. Basu A. (2011). Viral infection and neural stem/progenitor cell’s fate: implications in brain development and neurological disorders. *Neurochem. Int.* 59 357–366 10.1016/j.neuint.2011.02.020 21354238

[B69] de Groot R. H. Stein A. D. Jolles J. van Boxtel M. P. Blauw G.-J. van de Bor M. (2011). Prenatal famine exposure and cognition at age 59 years. *Int. J. Epidemiol.* 40 327–337 10.1093/ije/dyq261 21247885 PMC3066429

[B70] de Kloet E. R. Claessens S. E. F. Kentrop J. (2014). Context modulates outcome of perinatal glucocorticoid action in the brain. *Front. Endocrinol. (Lausanne)* 5:100 10.3389/fendo.2014.00100 PMC4088189 25071717

[B71] de Rooij S. R. Wouters H. Yonker J. E. Painter R. C. Roseboom T. J. (2010). Prenatal undernutrition and cognitive function in late adulthood. *Proc. Natl. Acad. Sci. U.S.A.* 107 16881–16886 10.1073/pnas.1009459107 20837515 PMC2947913

[B72] Desai M. Gayle D. A. Casillas E. Boles J. Ross M. G. (2009). Early undernutrition attenuates the inflammatory response in adult rat offspring. *J. Matern. Fetal Neonatal Med.* 22 571–575 10.1080/14767050902874105 19488945

[B73] de Souza A. S. Fernandes F. S. do Carmo M. D. G. T. (2011). Effects of maternal malnutrition and postnatal nutritional rehabilitation on brain fatty acids, learning, and memory. *Nutr. Rev.* 69 132–144 10.1111/j.1753-4887.2011.00374.x 21348877

[B74] Dimatelis J. J. Pillay N. S. Mutyaba A. K. Russell V. A. Daniels W. M. U. Stein D. J. (2012). Early maternal separation leads to down-regulation of cytokine gene expression. *Metab. Brain Dis.* 27 393–397 10.1007/s11011-012-9304-z 22527996

[B75] Dinel A. Joffre C. Trifilieff P. Aubert A. Foury A. Le Ruyet P. (2014). Inflammation early in life is a vulnerability factor for emotional behavior at adolescence and for lipopolysaccharide-induced spatial memory and neurogenesis alteration at adulthood. *J. Neuroinflammation* 11:155 10.1186/s12974-014-0155-x PMC4172903 25224537

[B76] Diz-Chaves Y. (2011). Ghrelin, appetite regulation, and food reward: interaction with chronic stress. *Int. J. Pept.* 2011:898450 10.1155/2011/898450 PMC3178114 21949667

[B77] Diz-Chaves Y. Astiz M. Bellini M. J. Garcia-Segura L. M. (2013). Prenatal stress increases the expression of proinflammatory cytokines and exacerbates the inflammatory response to LPS in the hippocampal formation of adult male mice. *Brain Behav. Immun.* 28 196–206 10.1016/j.bbi.2012.11.013 23207108

[B78] Diz-Chaves Y. Pernía O. Carrero P. Garcia-Segura L. M. (2012). Prenatal stress causes alterations in the morphology of microglia and the inflammatory response of the hippocampus of adult female mice. *J. Neuroinflammation* 9:71 10.1186/1742-2094-9-71 PMC3409032 22520439

[B79] Doosti M.-H. Bakhtiari A. Zare P. Amani M. Majidi-Zolbanin N. Babri S. (2013). Impacts of early intervention with fluoxetine following early neonatal immune activation on depression-like behaviors and body weight in mice. *Prog. Neuropsychopharmacol. Biol. Psychiatry* 43 55–65 10.1016/j.pnpbp.2012.12.003 23270703

[B80] Dranovsky A. Hen R. (2006). Hippocampal neurogenesis: regulation by stress and antidepressants. *Biol. Psychiatry* 59 1136–1143 10.1016/j.biopsych.2006.03.082 16797263 PMC7537828

[B81] Ekdahl C. T. (2012). Microglial activation – tuning and pruning adult neurogenesis. *Front. Pharmacol.* 3:41 10.3389/fphar.2012.00041 PMC3297835 22408626

[B82] Ekdahl C. T. Kokaia Z. Lindvall O. (2009). Brain inflammation and adult neurogenesis: the dual role of microglia. *Neuroscience* 158 1021–1029 10.1016/j.neuroscience.2008.06.052 18662748

[B83] Engelhardt B. (2003). Development of the blood-brain barrier. *Cell Tissue Res.* 314 119–129 10.1007/s00441-003-0751-z 12955493

[B84] Eriksen W. Sundet J. M. Tambs K. (2009). Register data suggest lower intelligence in men born the year after flu pandemic. *Ann. Neurol.* 66 284–289 10.1002/ana.21702 19798723

[B85] Fabricius K. Wörtwein G. Pakkenberg B. (2008). The impact of maternal separation on adult mouse behaviour and on the total neuron number in the mouse hippocampus. *Brain Struct. Funct.* 212 403–416 10.1007/s00429-007-0169-6 18200448 PMC2226080

[B86] Fedorova I. Hussein N. Baumann M. H. Di Martino C. Salem N. (2009). An n-3 fatty acid deficiency impairs rat spatial learning in the Barnes maze. *Behav. Neurosci.* 123 196–205 10.1037/a0013801 19170444

[B87] Feldman R. Singer M. Zagoory O. (2010). Touch attenuates infants’ physiological reactivity to stress. *Dev. Sci.* 13 271–278 10.1111/j.1467-7687.2009.00890.x 20136923

[B88] Fenoglio K. A. Brunson K. L. Baram T. Z. (2006). Hippocampal neuroplasticity induced by early-life stress: functional and molecular aspects. *Front. Neuroendocrinol.* 27:180–192 10.1016/j.yfrne.2006.02.001 16603235 PMC2937188

[B89] Fernández-Riejos P. Najib S. Santos-Alvarez J. Martín-Romero C. Pérez-Pérez A. González-Yanes C. (2010). Role of leptin in the activation of immune cells. *Mediators Inflamm.* 2010:568343 10.1155/2010/568343 PMC2846344 20368778

[B90] Francis D. D. Champagne F. C. Meaney M. J. (2000). Variations in maternal behaviour are associated with differences in oxytocin receptor levels in the rat. *J. Neuroendocrinol.* 12 1145–1148 10.1046/j.1365-2826.2000.00599.x 11106970

[B91] Francis D. D. Diorio J. Plotsky P. M. Meaney M. J. (2002). Environmental enrichment reverses the effects of maternal separation on stress reactivity. *J. Neurosci.* 22 7840–7843. 12223535 10.1523/JNEUROSCI.22-18-07840.2002 PMC6758090

[B92] Francis D. D. Meaney M. J. (1999). Maternal care and the development of stress responses. *Curr. Opin. Neurobiol.* 9 128–134 10.1016/S0959-4388(99)80016-6 10072372

[B93] Fukuda A. Fukuda H. Swanpalmer J. Hertzman S. Lannering B. Marky I. (2005). Age-dependent sensitivity of the developing brain to irradiation is correlated with the number and vulnerability of progenitor cells. *J. Neurochem.* 92 569–584 10.1111/j.1471-4159.2004.02894.x 15659227

[B94] Galic M. A. Riazi K. Henderson A. K. Tsutsui S. Pittman Q. J. (2009). Viral-like brain inflammation during development causes increased seizure susceptibility in adult rats. *Neurobiol. Dis.* 36 343–351 10.1016/j.nbd.2009.07.025 19660546 PMC3526656

[B95] Garden G. A. (2013). Epigenetics and the modulation of neuroinflammation. *Neurotherapeutics* 10 782–788 10.1007/s13311-013-0207-4 23963788 PMC3805872

[B96] Gebara E. Sultan S. Kocher-Braissant J. Toni N. (2013). Adult hippocampal neurogenesis inversely correlates with microglia in conditions of voluntary running and aging. *Front. Neurosci.* 7:145 10.3389/fnins.2013.00145 PMC3747329 23970848

[B97] Gómez-González B. Escobar A. (2009a). Altered functional development of the blood–brain barrier after early life stress in the rat. *Brain Res. Bull.* 79 376–387 10.1016/j.brainresbull.2009.05.012 19463912

[B98] Gómez-González B. Escobar A. (2009b). Prenatal stress alters microglial development and distribution in postnatal rat brain. *Acta Neuropathol.* 119 303–315 10.1007/s00401-009-0590-4 19756668

[B99] Gould E. Tanapat P. Rydel T. Hastings N. (2000). Regulation of hippocampal neurogenesis in adulthood. *Biol. Psychiatry* 48 715–720 10.1016/S0006-3223(00)01021-0 11063968

[B100] Green H. F. Nolan Y. M. (2014). Inflammation and the developing brain: consequences for hippocampal neurogenesis and behavior. *Neurosci. Biobehav. Rev.* 40 1–15 10.1016/j.neubiorev.2014.01.004 24462889

[B101] Greter M. Merad M. (2013). Regulation of microglia development and homeostasis. *Glia* 61 121–127 10.1002/glia.22408 22927325

[B102] Halassa M. M. Florian C. Fellin T. Munoz J. R. Lee S.-Y. Abel T. (2009). Astrocytic modulation of sleep homeostasis and cognitive consequences of sleep loss. *Neuron* 61 213–219 10.1016/j.neuron.2008.11.024 19186164 PMC2673052

[B103] Harré E. M. Galic M. A. Mouihate A. Noorbakhsh F. Pittman Q. J. (2008). Neonatal inflammation produces selective behavioural deficits and alters N-methyl-d-aspartate receptor subunit mRNA in the adult rat brain. *Eur. J. Neurosci.* 27 644–653 10.1111/j.1460-9568.2008.06031.x 18279317 PMC3547975

[B104] Hedges D. W. Woon F. L. (2011). Early-life stress and cognitive outcome. *Psychopharmacology (Berl.)* 214 121–130 10.1007/s00213-010-2090-6 21107538

[B105] Heijmans B. T. Tobi E. W. Stein A. D. Putter H. Blauw G. J. Susser E. S. (2008). Persistent epigenetic differences associated with prenatal exposure to famine in humans. *Proc. Natl. Acad. Sci. U.S.A.* 105 17046–17049 10.1073/pnas.0806560105 18955703 PMC2579375

[B106] Heim C. Binder E. B. (2012). Current research trends in early life stress and depression: review of human studies on sensitive periods, gene–environment interactions, and epigenetics. *Exp. Neurol.* 233 102–111 10.1016/j.expneurol.2011.10.032 22101006

[B107] Hulshof H. J. Novati A. Sgoifo A. Luiten P. G. den Boer J. A. Meerlo P. (2011). Maternal separation decreases adult hippocampal cell proliferation and impairs cognitive performance but has little effect on stress sensitivity and anxiety in adult Wistar rats. *Behav. Brain Res.* 216 552–560 10.1016/j.bbr.2010.08.038 20816703

[B108] Huot R. L. Plotsky P. M. Lenox R. H. McNamara R. K. (2002). Neonatal maternal separation reduces hippocampal mossy fiber density in adult Long Evans rats. *Brain Res.* 950 52–63 10.1016/S0006-8993(02)02985-2 12231228

[B109] Innis S. M. (2008). Dietary omega 3 fatty acids and the developing brain. *Brain Res.* 1237 35–43 10.1016/j.brainres.2008.08.078 18789910

[B110] Ivy A. S. Brunson K. L. Sandman C. Baram T. Z. (2008). Dysfunctional nurturing behavior in rat dams with limited access to nesting material: a clinically relevant model for early-life stress. *Neuroscience* 154 1132–1142 10.1016/j.neuroscience.2008.04.019 18501521 PMC2517119

[B111] Ivy A. S. Rex C. S. Chen Y. Dubé C. Maras P. M. Grigoriadis D. E. (2010). Hippocampal dysfunction and cognitive impairments provoked by chronic early-life stress involve excessive activation of CRH receptors. *J. Neurosci.* 30 13005–13015 10.1523/JNEUROSCI.1784-10.2010 20881118 PMC2991143

[B112] Iwasa T. Matsuzaki T. Kinouchi R. Fujisawa S. Murakami M. Kiyokawa M. (2010). Neonatal LPS injection alters the body weight regulation systems of rats under non-stress and immune stress conditions. *Int. J. Dev. Neurosci.* 28 119–124 10.1016/j.ijdevneu.2009.08.015 19733650

[B113] Järlestedt K. Naylor A. S. Dean J. Hagberg H. Mallard C. (2013). Decreased survival of newborn neurons in the dorsal hippocampus after neonatal LPS exposure in mice. *Neuroscience* 253 21–28 10.1016/j.neuroscience.2013.08.040 23994184 PMC3824076

[B114] Kamphuis P. Gardoni F. Kamal A. Croiset G. Bakker J. M. Cattabeni F. (2003). Long-lasting effects of neonatal dexamethasone treatment on spatial learning and hippocampal synaptic plasticity. Involvement of the NMDA receptor complex. *FASEB J.* 17 911–913 10.1096/fj.02-0333fje 12626441

[B115] Kaplan G. A. Turrell G. Lynch J. W. Everson S. A. Helkala E. L. Salonen J. T. (2001). Childhood socioeconomic position and cognitive function in adulthood. *Int. J. Epidemiol.* 30 256–263 10.1093/ije/30.2.256 11369724

[B116] Katz H. B. Davies C. A. Dobbing J. (1982). Effects of undernutrition at different ages early in Llife and later environmental complexity on parameters of the cerebrum and hippocampus in rats. *J. Nutr.* 112 1362–1368. 7097351 10.1093/jn/112.7.1362

[B117] Kelly D. Coutts A. G. P. (2000). Early nutrition and the development of immune function in the neonate. *Proc. Nutr. Soc.* 59 177–185 10.1017/S0029665100000197 10946785

[B118] Kempermann G. Jessberger S. Steiner B. Kronenberg G. (2004). Milestones of neuronal development in the adult hippocampus. *Trends Neurosci.* 27 447–452 10.1016/j.tins.2004.05.013 15271491

[B119] Kempermann G. Kuhn H. G. Gage F. H. (1997). More hippocampal neurons in adult mice living in an enriched environment. *Nature* 386 493–495 10.1038/386493a0 9087407

[B120] Kohman R. A. Rhodes J. S. (2013). Neurogenesis, inflammation and behavior. *Brain Behav. Immun.* 27 22–32 10.1016/j.bbi.2012.09.003 22985767 PMC3518576

[B121] Kohman R. A. Tarr A. J. Day C. E. McLinden K. A. Boehm G. W. (2008). Influence of prenatal stress on behavioral, endocrine, and cytokine responses to adulthood bacterial endotoxin exposure. *Behav. Brain Res.* 193 257–268 10.1016/j.bbr.2008.06.004 18590773

[B122] Korosi A. Baram T. Z. (2010). Plasticity of the stress response early in life: mechanisms and significance. *Dev. Psychobiol.* 52 661–670 10.1002/dev.20490 20862706 PMC3203734

[B123] Korosi A. Naninck E. F. G. Oomen C. A. Schouten M. Krugers H. Fitzsimons C. (2012). Early-life stress mediated modulation of adult neurogenesis and behavior. *Behav. Brain Res.* 227 400–409 10.1016/j.bbr.2011.07.037 21821065

[B124] Korosi A. Shanabrough M. McClelland S. Liu Z.-W. Borok E. Gao X.-B. (2010). Early-life experience reduces excitation to stress-responsive hypothalamic neurons and reprograms the expression of corticotropin-releasing hormone. *J. Neurosci.* 30 703–713 10.1523/JNEUROSCI.4214-09.2010 20071535 PMC2822406

[B125] Kumar G. Jones N. C. Morris M. J. Rees S. O’Brien T. J. Salzberg M. R. (2011). Early life stress enhancement of limbic epileptogenesis in adult rats: mechanistic insights. *PLoS ONE* 6:e24033 10.1371/journal.pone.0024033 PMC3177819 21957442

[B126] Lamptey M. S. Walker B. L. (1978). Learning behavior and brain lipid composition in rats subjected to essential fatty acid deficiency during gestation, lactation and growth. *J. Nutr.* 108 358–367. 627910 10.1093/jn/108.3.358

[B127] Laus M. F. Vales L. D. M. F. Costa T. M. B. Almeida S. S. (2011). Early postnatal protein-calorie malnutrition and cognition: a review of human and animal studies. *Int. J. Environ. Res. Public Health* 8 590–612 10.3390/ijerph8020590 21556206 PMC3084481

[B128] Lei X. Zhang W. Liu T. Xiao H. Liang W. Xia W. (2013). Perinatal supplementation with omega-3 polyunsaturated fatty acids improves sevoflurane-induced neurodegeneration and memory impairment in neonatal rats. *PLoS ONE* 8:e70645 10.1371/journal.pone.0070645 PMC3742769 23967080

[B129] Leventopoulos M. Rueedi-Bettschen D. Knuesel I. Feldon J. Pryce C. R. Opacka-Juffry J. (2007). Long-term effects of early life deprivation on brain glia in Fischer rats. *Brain Res.* 1142 119–126 10.1016/j.brainres.2007.01.039 17306230

[B130] Levine S. Huchton D. M. Wiener S. G. Rosenfeld P. (1991). Time course of the effect of maternal deprivation on the hypothalamic-pituitary-adrenal axis in the infant rat. *Dev. Psychobiol.* 24 547–558 10.1002/dev.420240803 1773913

[B131] Libbey J. Sweeten T. McMahon W. Fujinami R. (2005). Autistic disorder and viral infections. *J. Neurovirol.* 11 1–10 10.1080/13550280590900553 15804954

[B132] Lim S.-Y. Hoshiba J. Moriguchi T. Salem N. (2005). N-3 fatty acid deficiency induced by a modified artificial rearing method leads to poorer performance in spatial learning tasks. *Pediatr. Res.* 58 741–748 10.1203/01.PDR.0000180547.46725.CC 16189203

[B133] Lindqvist A. Mohapel P. Bouter B. Frielingsdorf H. Pizzo D. Brundin P. (2006). High-fat diet impairs hippocampal neurogenesis in male rats. *Eur. J. Neurol.* 13 1385–1388 10.1111/j.1468-1331.2006.01500.x 17116226

[B134] Liu B. Zupan B. Laird E. Klein S. Gleason G. Bozinoski M. (2014). Maternal hematopoietic TNF, via milk chemokines, programs hippocampal development and memory. *Nat. Neurosci.* 17 97–105 10.1038/nn.3596 24292233 PMC6169993

[B135] Liu D. Diorio J. Tannenbaum B. Caldji C. Francis D. Freedman A. (1997). Maternal care, hippocampal glucocorticoid receptors, and hypothalamic-pituitary-adrenal responses to stress. *Science* 277 1659–1662 10.1126/science.277.5332.1659 9287218

[B136] Llorente R. Arranz L. Marco E.-M. Moreno E. Puerto M. Guaza C. (2007). Early maternal deprivation and neonatal single administration with a cannabinoid agonist induce long-term sex-dependent psychoimmunoendocrine effects in adolescent rats. *Psychoneuroendocrinology* 32 636–650 10.1016/j.psyneuen.2007.04.002 17553622

[B137] Loi M. Koricka S. Lucassen P. J. Joëls M. (2014). Age- and sex-dependent effects of early life stress on hippocampal neurogenesis. *Front. Endocrinol.* 5:13 10.3389/fendo.2014.00013 PMC3929839 24600436

[B138] Lucas A. (1998). Programming by early nutrition: an experimental approach. *J. Nutr.* 128 401S–406S. 9478036 10.1093/jn/128.2.401S

[B139] Lucassen P. J. Meerlo P. Naylor A. S. Van Dam A. M. Dayer A. G. Fuchs E. (2010). Regulation of adult neurogenesis by stress, sleep disruption, exercise and inflammation: implications for depression and antidepressant action. *Eur. Neuropsychopharmacol.* 20 1–17 10.1016/j.euroneuro.2009.08.003 19748235

[B140] Lucassen P. J. Naninck E. F. G. van Goudoever J. B. Fitzsimons C. Joëls M. Korosi A. (2013). Perinatal programming of adult hippocampal structure and function; emerging roles of stress, nutrition and epigenetics. *Trends Neurosci.* 36 621–631 10.1016/j.tins.2013.08.002 23998452

[B141] Macrì S. Mason G. J. Wurbel H. (2004). Dissociation in the effects of neonatal maternal separations on maternal care and the offspring’s HPA and fear responses in rats. *Eur. J. Neurosci.* 20 1017–1024 10.1111/j.1460-9568.2004.03541.x 15305870

[B142] Madore C. Nadjar A. Delpech J.-C. Sere A. Aubert A. Portal C. (2014). Nutritional n-3 PUFAs deficiency during perinatal periods alters brain innate immune system and neuronal plasticity-associated genes. *Brain Behav. Immun.* 41 22–31 10.1016/j.bbi.2014.03.021 24735929

[B143] Marques A. H. O’Connor T. G. Roth C. Susser E. Bjørke-Monsen A.-L. (2013). The influence of maternal prenatal and early childhood nutrition and maternal prenatal stress on offspring immune system development and neurodevelopmental disorders. *Front. Neurosci.* 7:120 10.3389/fnins.2013.00120 PMC3728489 23914151

[B144] Martinez Y. Diaz-Cintra S. Leon-Jacinto U. Aguilar-Vazquez A. Medina A. C. Quirarte G. L. (2009). Effects of postnatal malnutrition and senescence on learning, long-term memory, and extinction in the rat. *Behav. Brain Res.* 203 48–53 10.1016/j.bbr.2009.04.016 19389427

[B145] Maselko J. Kubzansky L. Lipsitt L. Buka S. L. (2011). Mother’s affection at 8 months predicts emotional distress in adulthood. *J. Epidemiol. Community Health* 65 621–625 10.1136/jech.2009.097873 20660942 PMC3118641

[B146] Matos R. Orozco-Solis R. de Souza S. L. (2011). Nutrient restriction during early life reduces cell proliferation in the hippocampus at adulthood but does not impair the neuronal differentiation process of the new generated cells. *Neuroscience* 196 16–24 10.1016/j.neuroscience.2011.08.071 21930191

[B147] McMillen I. C. MacLaughlin S. M. Muhlhausler B. S. Gentili S. Duffield J. L. Morrison J. L. (2008). Developmental origins of adult health and disease: the role of periconceptional and foetal nutrition. *Basic Clin. Pharmacol. Toxicol.* 102 82–89 10.1111/j.1742-7843.2007.00188.x 18226059

[B148] McNamara R. K. Carlson S. E. (2006). Role of omega-3 fatty acids in brain development and function: potential implications for the pathogenesis and prevention of psychopathology. *Prostaglandins Leukot. Essent. Fatty Acids* 75 329–349 10.1016/j.plefa.2006.07.010 16949263

[B149] Meck W. H. Williams C. L. (1999). Choline supplementation during prenatal development reduces proactive interference in spatial memory. *Dev. Brain Res.* 118 51–59 10.1016/S0165-3806(99)00105-4 10611503

[B150] Mela V. Llorente-Berzal Á. Díaz F. Argente J. Viveros M.-P. Chowen J. A. (2012). Maternal deprivation exacerbates the response to a high fat diet in a sexually dimorphic manner. *PLoS ONE* 7:e48915 10.1371/journal.pone.0048915 PMC3492147 23145019

[B151] Miller A. A. Spencer S. J. (2014). Obesity and neuroinflammation: a pathway to cognitive impairment. *Brain Behav. Immun.* 42 10–21 10.1016/j.bbi.2014.04.001 24727365

[B152] Miller B. J. Culpepper N. Rapaport M. H. Buckley P. (2013). Prenatal inflammation and neurodevelopment in schizophrenia: a review of human studies. *Prog. Neuropsychopharmacol. Biol. Psychiatry* 42 92–100 10.1016/j.pnpbp.2012.03.010 22510462

[B153] Miller G. E. Chen E. Fok A. K. Walker H. Lim A. Nicholls E. F. (2009). Low early-life social class leaves a biological residue manifested by decreased glucocorticoid and increased proinflammatory signaling. *Proc. Natl. Acad. Sci. U.S.A.* 106 14716–14721 10.1073/pnas.0902971106 19617551 PMC2732821

[B154] Mirescu C. Peters J. D. Gould E. (2004). Early life experience alters response of adult neurogenesis to stress. *Nat. Neurosci.* 7 841–846 10.1038/nn1290 15273691

[B155] Monk C. Georgieff M. K. Osterholm E. A. (2013). Research review: maternal prenatal distress and poor nutrition - mutually influencing risk factors affecting infant neurocognitive development. *J. Child Psychol. Psychiatry* 54 115–130 10.1111/jcpp.12000 23039359 PMC3547137

[B156] Morris M. S. (2003). Homocysteine and Alzheimer’s disease. *Lancet Neurol.* 2 425–428 10.1016/S1474-4422(03)00438-1 12849121

[B157] Mueller S. C. Maheu F. S. Dozier M. Peloso E. Mandell D. Leibenluft E. (2010). Early-life stress is associated with impairment in cognitive control in adolescence: an fMRI study. *Neuropsychologia* 48 3037–3044 10.1016/j.neuropsychologia.2010.06.013 20561537 PMC2916226

[B158] Murgatroyd C. Patchev A. V. Wu Y. Micale V. Bockmühl Y. Fischer D. (2009). Dynamic DNA methylation programs persistent adverse effects of early-life stress. *Nat. Neurosci.* 12 1559–1566 10.1038/nn.2436 19898468

[B159] Musaelyan K. Egeland M. Fernandes C. Pariante C. M. Zunszain P. A. Thuret S. (2014). Modulation of adult hippocampal neurogenesis by early-life environmental challenges triggering immune activation. *Neural Plast.* 2014 194396 10.1155/2014/194396 PMC4033517 24891958

[B160] Nair A. Vadodaria K. C. Banerjee S. B. Benekareddy M. Dias B. G. Duman R. S. (2007). Stressor-specific regulation of distinct brain-derived neurotrophic factor transcripts and cyclic AMP response element-binding protein expression in the postnatal and adult rat hippocampus. *Neuropsychopharmacology* 32 1504–1519 10.1038/sj.npp.1301276 17164818

[B161] Naninck E. F. G. Hoeijmakers L. Kakava-Georgiadou N. Meesters A. Lazic S. E. Lucassen P. J. (2014). Chronic early-life stress alters developmental and adult neurogenesis and impairs cognitive function in mice. *Hippocampus.* 10.1002/hipo.22374 [Epub ahead of print]. 25269685

[B162] Naninck E. F. G. Lucassen P. J. Korosi A. (2013). “Consequences of early-life experiences on cognition and emotion: a role for nutrition and epigenetic mechanisms,” in *Molecular Psychology*, ed. Canli T. (Oxford Handbooks Online) 10.1093/oxfordhb/9780199753888.013.003

[B163] Navarro-Quiroga I. Hernandez-Valdes M. Lin S. L. Naegele J. R. (2006). Postnatal cellular contributions of the hippocampus subventricular zone to the dentate gyrus, corpus callosum, fimbria, and cerebral cortex. *J. Comp. Neurol.* 497 833–845 10.1002/cne.21037 16786555

[B164] Nelson C. A. Zeanah C. H. Fox N. A. Marshall P. J. Smyke A. T. Guthrie D. (2007). Cognitive recovery in socially deprived young children: the Bucharest early intervention project. *Science* 318 1937–1940 10.1126/science.1143921 18096809

[B165] Niculescu M. D. Lupu D. S. Craciunescu C. N. (2011). Maternal α-linolenic acid availability during gestation and lactation alters the postnatal hippocampal development in the mouse offspring. *Int. J. Dev. Neurosci.* 29 795–802 10.1016/j.ijdevneu.2011.09.006 21964326 PMC4324752

[B166] Nilsson M. Perfilieva E. Johansson U. Orwar O. Eriksson P. S. (1999). Enriched environment increases neurogenesis in the adult rat dentate gyrus and improves spatial memory. *J. Neurobiol.* 39 569–578 10.1002/(SICI)1097-4695(19990615)39:4<569::AID-NEU10>3.0.CO;2-F 10380078

[B167] O’Connor T. G. Moynihan J. A. Caserta M. T. (2014). Annual research review: the neuroinflammation hypothesis for stress and psychopathology in children–developmental psychoneuroimmunology. *J. Child Psychol. Psychiatry* 55 615–631 10.1111/jcpp.12187 24372371 PMC4029900

[B168] Oitzl M. S. Workel J. O. Fluttert M. Frosch F. de Kloet E. R. (2000). Maternal deprivation affects behaviour from youth to senescence: amplification of individual differences in spatial learning and memory in senescent Brown Norway rats. *Eur. J. Neurosci.* 12 3771–3780 10.1046/j.1460-9568.2000.00231.x 11029647

[B169] Olah M. Ping G. De Haas A. H. Brouwer N. Meerlo P. Van Der Zee E. A. (2009). Enhanced hippocampal neurogenesis in the absence of microglia T cell interaction and microglia activation in the murine running wheel model. *Glia* 57 1046–1061 10.1002/glia.20828 19115394

[B170] Oomen C. A. Girardi C. E. N. Cahyadi R. Verbeek E. C. Krugers H. Joëls M. (2009). Opposite effects of early maternal deprivation on neurogenesis in male versus female rats. *PLoS ONE* 4:e3675 10.1371/journal.pone.0003675 PMC2629844 19180242

[B171] Oomen C. A. Soeters H. Audureau N. Vermunt L. van Hasselt F. N. Manders E. M. M. (2010). Severe early life stress hampers spatial learning and neurogenesis, but improves hippocampal synaptic plasticity and emotional learning under high-stress conditions in adulthood. *J. Neurosci.* 30 6635–6645 10.1523/JNEUROSCI.0247-10.2010 20463226 PMC6632559

[B172] Oomen C. A. Soeters H. Audureau N. Vermunt L. van Hasselt F. N. Manders E. M. M. (2011). Early maternal deprivation affects dentate gyrus structure and emotional learning in adult female rats. *Psychopharmacology (Berl.)* 214 249–260 10.1007/s00213-010-1922-8 20589492 PMC3045507

[B173] Orr A. G. Sharma A. Binder N. B. Miller A. H. Pearce B. D. (2010). Interleukin-1 mediates long-term hippocampal dentate granule cell loss following postnatal viral infection. *J. Mol. Neurosci.* 41 89–96 10.1007/s12031-009-9293-5 19774496

[B174] Ortega A. Jadeja V. Zhou H. (2011). Postnatal development of lipopolysaccharide-induced inflammatory response in the brain. *Inflamm. Res.* 60 175–185 10.1007/s00011-010-0252-y 20865294

[B175] Palacio A. Lopez M. Perez-Bravo F. Monkeberg F. Schlesinger L. (2002). Leptin levels are associated with immune response in malnourished infants. *J. Clin. Endocrinol. Metab.* 87 3040–3046 10.1210/jcem.87.7.8636 12107198

[B176] Palmer A. C. (2011). Nutritionally mediated programming of the developing immune system. *Adv. Nutr.* 2 377–395 10.3945/an.111.000570 22332080 PMC3183589

[B177] Pan W. Kastin A. J. (1999). Penetration of neurotrophins and cytokines across the blood–brain/blood–spinal cord barrier. *Adv. Drug Deliv. Rev.* 36 291–298 10.1016/S0169-409X(98)00086-6 10837721

[B178] Parpura V. Heneka M. T. Montana V. Oliet S. H. R. Schousboe A. Haydon P. G. (2012). Glial cells in (patho)physiology. *J. Neurochem.* 121 4–27 10.1111/j.1471-4159.2012.07664.x 22251135 PMC3304021

[B179] Parylak S. L. Deng W. Gage F. H. (2014). Mother’s milk programs offspring’s cognition. *Nat. Neurosci.* 17 8–9 10.1038/nn.3611 24369371

[B180] Pearce B. D. Steffensen S. C. Paoletti A. D. Henriksen S. J. Buchmeier M. J. (1996). Persistent dentate granule cell hyperexcitability after neonatal infection with lymphocytic choriomeningitis virus. *J. Neurosci.* 16 220–228. 8613788 10.1523/JNEUROSCI.16-01-00220.1996 PMC6578731

[B181] Pleasure S. J. Collins A. E. Lowenstein D. H. (2000). Unique expression patterns of cell fate molecules delineate sequential stages of dentate gyrus development. *J. Neurosci.* 20 6095–6105. 10934259 10.1523/JNEUROSCI.20-16-06095.2000 PMC6772596

[B182] Plotsky P. M. Meaney M. J. (1993). Early, postnatal experience alters hypothalamic corticotropin-releasing factor (CRF) mRNA, median eminence CRF content and stress-induced release in adult rats. *Mol. Brain Res.* 18 195–200 10.1016/0169-328X(93)90189-V 8497182

[B183] Prado E. L. Dewey K. G. (2014). Nutrition and brain development in early life. *Nutr. Rev.* 72 267–284 10.1111/nure.12102 24684384

[B184] Pryce C. R. Bettschen D. Feldon J. (2001). Comparison of the effects of early handling and early deprivation on maternal care in the rat. *Dev. Psychobiol.* 38 239–251 10.1002/dev.1018 11319730

[B185] Rantakallio P. Jones P. Moring J. Von Wendt L. (1997). Association between central nervous system infections during childhood and adult onset schizophrenia and other psychoses: a 28-year follow-up. *Int. J. Epidemiol.* 26 837–843 10.1093/ije/26.4.837 9279617

[B186] Rice C. J. Sandman C. A. Lenjavi M. R. Baram T. Z. (2008). A novel mouse model for acute and long-lasting consequences of early life stress. *Endocrinology* 149 4892–4900 10.1210/en.2008-0633 18566122 PMC2582918

[B187] Roy S. Kale A. Dangat K. Sable P. Kulkarni A. Joshi S. (2012). Maternal micronutrients (folic acid and vitamin B(12)) and omega 3 fatty acids: implications for neurodevelopmental risk in the rat offspring. *Brain Dev.* 34 64–71 10.1016/j.braindev.2011.01.002 21300490

[B188] Sauder C. Wolfer D. P. Lipp H. P. Staeheli P. Hausmann J. (2001). Learning deficits in mice with persistent Borna disease virus infection of the CNS associated with elevated chemokine expression. *Behav. Brain Res.* 120 189–201 10.1016/S0166-4328(00)00370-3 11182167

[B189] Schmidt M. Enthoven L. Van Woezik J. H. G. Levine S. de Kloet E. R. Oitzl M. S. (2004). The Dynamics of the hypothalamic-pituitary-adrenal axis during maternal deprivation. *J. Neuroendocrinol.* 16 52–57 10.1111/j.1365-2826.2004.01123.x 14962076

[B190] Schmidt M. V. Wang X.-D. Meijer O. C. (2010). Early life stress paradigms in rodents: potential animal models of depression? *Psychopharmacology* 214 131–140 10.1007/s00213-010-2096-0 21086114

[B191] Schoderboeck L. Adzemovic M. Nicolussi E.-M. Crupinschi C. Hochmeister S. Fischer M.-T. (2009). The “window of susceptibility” for inflammation in the immature central nervous system is characterized by a leaky blood–brain barrier and the local expression of inflammatory chemokines. *Neurobiol. Dis.* 35 368–375 10.1016/j.nbd.2009.05.026 19520164 PMC3703512

[B192] Scholl T. O. Hediger M. L. Schall J. I. Khoo C. S. Fischer R. L. (1996). Dietary and serum folate: their influence on the outcome of pregnancy. *Am. J. Clin. Nutr.* 63 520–525. 8599315 10.1093/ajcn/63.4.520

[B193] Schwarz J. M. Bilbo S. D. (2012). Sex, glia, and development: interactions in health and disease. *Horm. Behav.* 62 243–253 10.1016/j.yhbeh.2012.02.018 22387107 PMC3374064

[B194] Schwarz J. M. Sholar P. W. Bilbo S. D. (2012). Sex differences in microglial colonization of the developing rat brain. *J. Neurochem.* 120 948–963 10.1111/j.1471-4159.2011.07630.x 22182318 PMC3296888

[B195] Seckl J. R. Meaney M. J. (2006). Glucocorticoid “programming” and PTSD risk. *Ann. N. Y. Acad. Sci.* 1071 351–378 10.1196/annals.1364.027 16891583

[B196] Shanks N. Lightman S. L. (2001). The maternal-neonatal neuro-immune interface: are there long-term implications for inflammatory or stress-related disease? *J. Clin. Invest.* 108 1567–1573 10.1172/JCI14592 11733549 PMC200999

[B197] Sharma A. Valadi N. Miller A. H. Pearce B. D. (2002). Neonatal viral infection decreases neuronal progenitors and impairs adult neurogenesis in the hippocampus. *Neurobiol. Dis.* 11 246–256 10.1006/nbdi.2002.0531 12505418

[B198] Sierra A. Abiega O. Shahraz A. Neumann H. (2013). Janus-faced microglia: beneficial and detrimental consequences of microglial phagocytosis. *Front. Cell. Neurosci.* 7:6 10.3389/fncel.2013.00006 PMC3558702 23386811

[B199] Sierra A. Beccari S. Diaz-Aparicio I. Encinas J. M. Comeau S. Tremblay M.-E. (2014). Surveillance, phagocytosis, and inflammation: how never-resting microglia influence adult hippocampal neurogenesis. *Neural Plast.* 2014:610343 10.1155/2014/610343 PMC3977558 24772353

[B200] Singer P. Shapiro H. Theilla M. Anbar R. Singer J. Cohen J. (2008). Anti-inflammatory properties of omega-3 fatty acids in critical illness: novel mechanisms and an integrative perspective. *Intensive Care Med.* 34 1580–1592 10.1007/s00134-008-1142-4 18461305

[B201] Slezak M. Pfrieger F. W. Soltys Z. (2006). Synaptic plasticity, astrocytes and morphological homeostasis. *J. Physiol. Paris* 99 84–91 10.1016/j.jphysparis.2005.12.082 16459062

[B202] Sominsky L. Walker A. K. Ong L. K. Tynan R. J. Walker F. R. Hodgson D. M. (2012). Increased microglial activation in the rat brain following neonatal exposure to a bacterial mimetic. *Behav. Brain Res.* 226 351–356 10.1016/j.bbr.2011.08.038 21907243

[B203] Spencer S. J. (2013). Perinatal nutrition programs neuroimmune function long-term: mechanisms and implications. *Front. Neurosci.* 7:144 10.3389/fnins.2013.00144 PMC3740243 23964195

[B204] Stanton M. E. Gutierrez Y. R. Levine S. (1988). Maternal deprivation potentiates pituitary-adrenal stress responses in infant rats. *Behav. Neurosci.* 102 692–700 10.1037/0735-7044.102.5.692 3196438

[B205] Steegers-Theunissen R. P. Obermann-Borst S. A. Kremer D. Lindemans J. Siebel C. Steegers E. A. (2009). Periconceptional maternal folic acid use of 400 μg per day is related to increased methylation of the IGF2 gene in the very young child. *PLoS ONE* 4:e7845 10.1371/journal.pone.0007845 PMC2773848 19924280

[B206] Stevens S. E. Sonuga-Barke E. J. S. Kreppner J. M. Beckett C. Castle J. Colvert E. (2008). Inattention/overactivity following early severe institutional deprivation: presentation and associations in early adolescence. *J. Abnorm. Child Psychol.* 36 385–398 10.1007/s10802-007-9185-5 17965931

[B207] Suri D. Veenit V. Sarkar A. Thiagarajan D. Kumar A. Nestler E. J. (2013). Early stress evokes age-dependent biphasic changes in hippocampal neurogenesis, BDNF expression, and cognition. *Biol. Psychiatry* 73 658–666 10.1016/j.biopsych.2012.10.023 23237316 PMC4051354

[B208] Tozuka Y. Kumon M. Wada E. Onodera M. Mochizuki H. Wada K. (2010). Maternal obesity impairs hippocampal BDNF production and spatial learning performance in young mouse offspring. *Neurochem. Int.* 57 235–247 10.1016/j.neuint.2010.05.015 20538025

[B209] Tozuka Y. Wada E. Wada K. (2009). Diet-induced obesity in female mice leads to peroxidized lipid accumulations and impairment of hippocampal neurogenesis during the early life of their offspring. *FASEB J.* 23 1920–1934 10.1096/fj.08-124784 19158155

[B210] Troen A. M. Shea-Budgell M. Shukitt-Hale B. Smith D. E. Selhub J. Rosenberg I. H. (2008). B-vitamin deficiency causes hyperhomocysteinemia and vascular cognitive impairment in mice. *Proc. Natl. Acad. Sci. U.S.A.* 105 12474–12479 10.1073/pnas.0805350105 18711131 PMC2517600

[B211] Tsigos C. Chrousos G. P. (2002). Hypothalamic-pituitary-adrenal axis, neuroendocrine factors and stress. *J. Psychosom. Res.* 53 865–871 10.1016/S0022-3999(02)00429-4 12377295

[B212] Valadares C. T. Fukuda M. T. H. Francolin-Silva A. L. Hernandes A. S. Almeida S. S. (2010). Effects of postnatal protein malnutrition on learning and memory procedures. *Nutr. Neurosci.* 13 274–282 10.1179/147683010X12611460764769 21040625

[B213] Valladolid-Acebes I. Stucchi P. Cano V. Fernandez-Alfonso M. S. Merino B. Gil-Ortega M. (2011). High-fat diets impair spatial learning in the radial-arm maze in mice. *Neurobiol. Learn. Mem.* 95 80–85 10.1016/j.nlm.2010.11.007 21093599

[B214] Van Dam A. M. Brouns M. Louisse S. Berkenbosch F. (1992). Appearance of interleukin-1 in macrophages and in ramified microglia in the brain of endotoxin-treated rats: a pathway for the induction of non-specific symptoms of sickness? *Brain Res.* 588 291–296 10.1016/0006-8993(92)91588-6 1393581

[B215] Van Dam F. Van Gool W. A. (2009). Hyperhomocysteinemia and Alzheimer’s disease: a systematic review. *Arch. Gerontol. Geriatr.* 48 425–430 10.1016/j.archger.2008.03.009 18479766

[B216] van der Ree M. Tanis J. C. Van Braeckel K. N. J. A. Bos A. F. Roze E. (2011). Functional impairments at school age of preterm born children with late-onset sepsis. *Early Hum. Dev.* 87 821–826 10.1016/j.earlhumdev.2011.06.008 21752558

[B217] van Hasselt F. N. Cornelisse S. Zhang T. Y. Meaney M. J. Velzing E. H. Krugers H. J. (2012a). Adult hippocampal glucocorticoid receptor expression and dentate synaptic plasticity correlate with maternal care received by individuals early in life. *Hippocampus* 22 255–266 10.1002/hipo.20892 21240921

[B218] van Hasselt F. N. Tieskens J. M. Trezza V. Krugers H. J. Vanderschuren L. J. M. J. Joels M. (2012b). Within-litter variation in maternal care received by individual pups correlates with adolescent social play behavior in male rats. *Physiol. Behav.* 106 701–706 10.1016/j.physbeh.2011.12.007 22210522

[B219] Veena S. R. Krishnaveni G. V. Srinivasan K. Wills A. K. Muthayya S. Kurpad A. V. (2010). Higher maternal plasma folate but not vitamin B-12 concentrations during pregnancy are associated with better cognitive function scores in 9- to 10- year-old children in South India. *J. Nutr.* 140 1014–1022 10.3945/jn.109.118075 20335637 PMC3672847

[B220] Viviani B. Boraso M. Valero M. Gardoni F. Marco E.-M. Llorente R. (2013). Early maternal deprivation immunologically primes hippocampal synapses by redistributing interleukin-1 receptor type I in a sex dependent manner. *Brain Behav. Immun.* 35 135–143 10.1016/j.bbi.2013.09.008 24060584

[B221] Walker A. K. Nakamura T. Hodgson D. M. (2010). Neonatal lipopolysaccharide exposure alters central cytokine responses to stress in adulthood in Wistar rats. *Stress* 13 506–515 10.3109/10253890.2010.489977 20666652

[B222] Walker S. P. Grantham-McGregor S. M. Powell C. A. Chang S. M. (2000). Effects of growth restriction in early childhood on growth, IQ, and cognition at age 11 to 12 years and the benefits of nutritional supplementation and psychosocial stimulation. *J. Pediatr.* 137 36–41 10.1067/mpd.2000.106227 10891819

[B223] Wang K. C. Fan L. W. Kaizaki A. Pang Y. Cai Z. Tien L. T. (2013). Neonatal lipopolysaccharide exposure induces long-lasting learning impairment, less anxiety-like response and hippocampal injury in adult rats. *Neuroscience* 234 146–157 10.1016/j.neuroscience.2012.12.049 23298854 PMC3594355

[B224] Wang X.-D. Rammes G. Kraev I. Wolf M. Liebl C. Scharf S. H. (2011). Forebrain CRF1 modulates early-life stress-programmed cognitive deficits. *J. Neurosci.* 31 13625–13634 10.1523/JNEUROSCI.2259-11.2011 21940453 PMC3380621

[B225] Weaver I. C. G. Champagne F. A. Brown S. E. Dymov S. Sharma S. Meaney M. J. (2005). Reversal of maternal programming of stress responses in adult offspring through methyl supplementation: altering epigenetic marking later in life. *J. Neurosci.* 25 11045–11054 10.1523/JNEUROSCI.3652-05.2005 16306417 PMC6725868

[B226] Wei L. Simen A. Mane S. Kaffman A. (2012). Early life stress inhibits expression of a novel innate immune pathway in the developing hippocampus. *Neuropsychopharmacology* 37 567–580 10.1038/npp.2011.239 21993208 PMC3242319

[B227] Williamson L. L. Chao A. Bilbo S. D. (2012). Environmental enrichment alters glial antigen expression and neuroimmune function in the adult rat hippocampus. *Brain Behav. Immun.* 26 500–510 10.1016/j.bbi.2012.01.003 22281279 PMC3294275

[B228] Williamson L. L. Sholar P. W. Mistry R. S. Smith S. H. Bilbo S. D. (2011). Microglia and memory: modulation by early-life infection. *J. Neurosci.* 31 15511–15521 10.1523/JNEUROSCI.3688-11.2011 22031897 PMC3224817

[B229] Wolf C. Almli C. R. Finger S. Ryan S. Morgane P. J. (1986). Behavioral effects of severe and moderate early malnutrition. *Physiol. Behav.* 38 725–730 10.1016/0031-9384(86)90270-2 3823189

[B230] Yi S. J. Baram T. Z. (1994). Corticotropin-releasing hormone mediates the response to cold stress in the neonatal rat without compensatory enhancement of the peptide’s gene expression. *Endocrinology* 135 2364–2368 10.1210/endo.135.6.7988418 7988418 PMC3783019

[B231] Ziko I. De Luca S. Dinan T. Barwood J. M. Sominsky L. Cai G. (2014). Neonatal overfeeding alters hypothalamic microglial profiles and central responses to immune challenge long-term. *Brain Behav. Immun.* 41 32–43 10.1016/j.bbi.2014.06.014 24975592

[B232] Zimmerberg B. Foote H. E. Van Kempen T. A. (2009). Olfactory association learning and brain-derived neurotrophic factor in an animal model of early deprivation. *Dev. Psychobiol.* 51 333–344 10.1002/dev.20373 19308959

[B233] Zocher M. Czub S. Schulte-Mönting J. de la Torre J. C. Sander C. (2000). Alterations in neurotrophin and neurotrophin receptor gene expression patterns in the rat central nervous system following perinatal Borna disease virus infection. *J. Neurovirol.* 6 462–477 10.3109/13550280009091947 11175319

[B234] Zou C.-G. Zhao Y.-S. Gao S.-Y. Li S.-D. Cao X.-Z. Zhang M. (2010). Homocysteine promotes proliferation and activation of microglia. *Neurobiol. Aging* 31 2069–2079 10.1016/j.neurobiolaging.2008.11.007 19131143

